# Rational design and preclinical evaluation of elastin-like polypeptide micelle nanoparticles for drug delivery

**DOI:** 10.3389/fbioe.2026.1736210

**Published:** 2026-04-21

**Authors:** Abdulhakim Tofik, Yuwei Zhang, Cassandra Volpe, Jing Wang

**Affiliations:** Department of Chemical and Biological Engineering, Iowa State University, Ames, IA, United States

**Keywords:** biomaterial, drug delivery, elastin-like polypeptide, micelle, rational design

## Abstract

Elastin-like polypeptide (ELP) micelle nanoparticles have emerged as versatile and tunable platforms for drug delivery across diseases. These bio-inspired and thermo-responsive polymers typically produced recombinantly in *E.coli* or yeast offer great biocompatibility, low immunogenicity, and cost-effective production. Rationally designed amphiphilic diblock ELPs, ELP-drug/polymer/protein conjugates, and ELP-nucleic acid polyplexes can self-assemble into micelle or micelle-like nanoparticles at physiological temperatures. Similar to other nanoparticle drug delivery systems, ELP micelles can load a broad range of drugs, prolong systemic circulation, and enable controlled and sustained drug release. Notably, ELP micelles provide unique advantages for delivering protein and peptide drugs, as their conjugates with ELP can be recombinantly synthesized with a 100% conjugation efficiency, eliminating the need for chemical coupling. In this review, we will discuss the design principles of ELP micelle-based drug delivery systems and summarize their recent applications in cancer therapy and vaccine development.

## 1 Introduction

**FIGURE 1:**
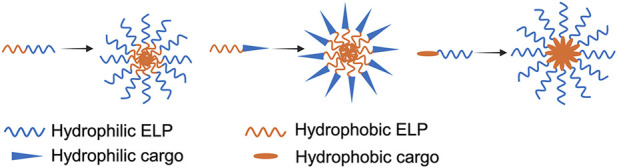
Schematic illustration showing self-assembly of diblock ELP and amphiphilic ELP-cargo conjugates into micelles at 37 °C. Each diblock ELPs contains a hydrophobic segment (T_t_ < 37 °C) and a hydrophilic segment (T_t_ > 37 °C).

Elastin-like polypeptides (ELPs) are a class of genetically engineered polypeptides with a sequence of (VPGXG)n, where the guest residue X in the pentapeptide can be any amino acid residue except proline (Meyer and Chilkoti, 1999; Urry, 1988). ELPs undergo a lower critical solution temperature (LCST) phase transition. Below the LCST, also referred to as the inverse transition temperature (T_t_), ELPs are fully soluble and adopts a disordered coil structure in aqueous solution. Above T_t_, they coacervate into β-spiral structure and form insoluble aggregates (Smits et al., 2015). This thermo-responsive phase transition is reversible and tunable by varying the identity of the guest residue and the number of pentapeptide repeats. Diblock ELP copolymers (ELP_BC_) composed of two segments with different T_t_ values—one above and one below 37 °C—can self-assemble into micelle nanoparticles at the physiological temperature. In these micelles, the ELP block with T_t_ < 37 °C forms the hydrophobic core, while the block with T_t_ > 37 °C forms the hydrophilic corona. Similarly, conjugation of water-insoluble small-molecule drugs or hydrophobic polymers to an ELP with T_t_ > 37 °C, or conjugation of water-soluble proteins, nucleic acids, or hydrophilic polymers to an ELP with T_t_ < 37 °C, can induce the formation of micelles or micelle-like nanoparticles at the physiological temperature (Figure 1). With well-validated biocompatibility and low immunogenicity, ELP-based micelle nanoparticles have been widely investigated as versatile and tunable platforms for drug delivery across a broad range of therapeutic applications (Jenkins et al., 2021; Jiang et al., 2023; van Strien et al., 2023). In this review, we will discuss the design principles of ELP micelle-based drug delivery systems and highlight their applications in cancer therapy and vaccine development, focusing on their efficacy in preclinical models.

## 2 Rational design of ELP micelles for drug delivery

### 2.1 ELP’s T_t_

**FIGURE 2:**
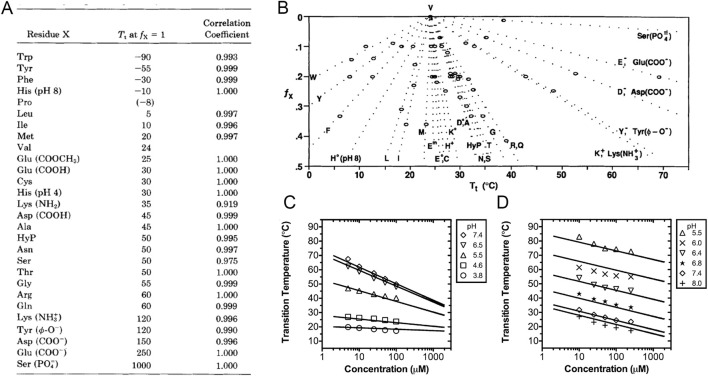
ELP’s T_t_ depends on guest residue composition, chain length, polymer concentration, and solution pH (for ELPs containing ionizable residues). **(A)** T_t_ for poly [f_v_ (VPGVG), f_x_ (VPGXG)] in PBS as a function of f_x_ (Urry et al., 1992). **(B)** T_t_ for poly [f_v_ (VPGVG), f_x_ (VPGXG)] at f_x_ = 1 (Urry et al., 1992). **(C,D)** T_t_ of (C) an acidic ELP and (D) a basic ELP at different polymer concentration and pH. The acidic ELP (pK_a_ = 5.29) had the sequence of (VPGXG)_160_, where X = V: I: E = 1: 3: 1. The basic ELP (pK_a_ = 6.22) had the sequence of (VPGXG)_120_, where X = V: H: G: A = 1: 2: 1: 1 (Mackay et al., 2010).

ELP’s T_t_ can be finely tuned by altering the guest residue and the number of pentapeptide repeats. Urry et al. demonstrated that increasing the mean hydrophobicity of the guest residue decreased ELP’s T_t_. The ELP used in their study was poly [f_x_ (VPGXG), f_v_ (VPGVG))], where f_x_ and f_v_ represent mole fractions of VPGXG and VPGVG, respectively, and f_x_ + f_v_ = 1. When f_x_ was fixed, the T_t_ of (VPGXG)_n_ varied with the hydrophobicity of the guest residue in the order: W < Y < F < H (pH 8) < L < I < M < V < H^+^, E, C < K < A, D < T, N, S < G < R, Q < K^+^, Y^−^, D^−^ < E^−^ (Figure 2A) (Urry et al., 1991a; Urry et al., 1992; Urry, 1993; Urry, 1997). When X = W, Y, F, H, L, or I—all residues more hydrophobic than V—an increase in f_x_ enhanced the overall hydrophobicity of ELPs, thereby reducing ELP’s T_t_. This effect was more pronounced in ELPs with inherently low T_t_ (i.e., high hydrophobicity). In contrast, when X = A, G or other polar residues more hydrophilic than V, an increase in f_x_ elevated T_t_, with the effect being more pronounced in ELPs with inherently high T_t_ (i.e., high hydrophilicity) (Figure 2B) (Urry et al., 1992). These findings were consistent with results reported by McDaniel and Meyer et al. using a similar mixed ELP of (VPGXG)_n_, where X consisted of a mixture of A and V or A, V and G, to examine the influence of guest residue composition on ELP’s T_t_ (McDaniel et al., 2013a; Meyer and Chilkoti, 2004).

ELP’s T_t_ is also influenced by chain length, polymer concentration, and environmental factors such as ion type and ionic strength. It has been well demonstrated that longer ELPs exhibit lower T_t_ values than shorter ones with the same pentapeptide sequences, and that T_t_ decreases with increasing polymer concentration or higher ionic strength (Meyer and Chilkoti, 2004) (Dai et al., 2021; Ribeiro et al., 2009). A mathematical model has been established to quantitatively describe the relationship between ELP’s T_t_, chain length, and polymer concentration (McDaniel et al., 2013a). The type of ions in the solution also affects ELP’s T_t_. The solubility of ELP proteins in various salt solutions follows the Hofmeister series, in which the anion sequence is: CO_3_^2-^ > SO_4_^2-^ > S_2_O_3_^2-^ > H_2_PO_4_^−^ > F^−^ > Cl^−^ > Br^−^, NO^3-^ > I^−^ > ClO_4_^−^ > SCN^−^. Kosmotropic anions (species to the left of Cl^−^) tend to “salt out” proteins by decreasing their solubility, whereas chaotropic ions (species to the right of Cl^−^) increase protein solubility. Cho et al. studied the T_t_ of two ELPs, (VPGVG)_120_ and (VPGXG)_120_, where X = V: A: G = 5: 2: 3 (sometimes described as X = V5A2G3), in 11 different sodium salts. They observed that kosmotropic anions such as CO_3_^2-^ and SO_4_^2-^ produced a more pronounced decrease in T_t_ compared with chaotropic anions like ClO_4_^−^ and Br^−^, and that T_t_ decreased further with increasing ion concentration. Interestingly, chaotropic anions such as SCN^−^ and I^−^ did not significantly reduce T_t_. Their analysis suggested that kosmotropic anions lower T_t_ by polarizing interfacial water molecules that hydrate the ELP amide groups, whereas chaotropic anions influence T_t_ primarily through surface tension effects (Cho et al., 2008).

**FIGURE 3:**
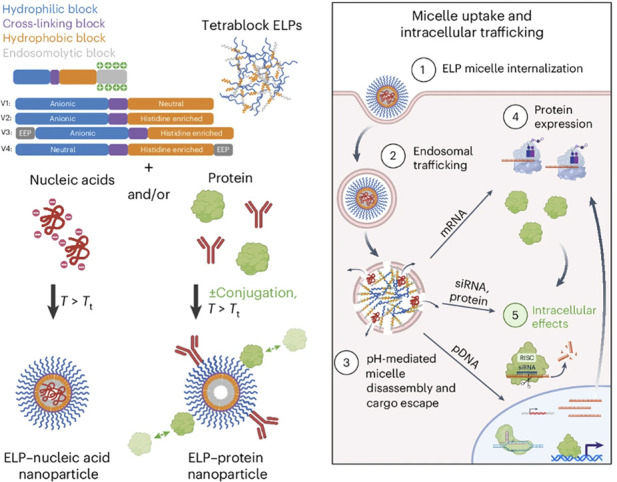
Schematic illustration of micelle-forming ELPs containing (from N terminus to C terminus) a hydrophilic block, cysteine-based crosslinking block, hydrophobic block and endosomolytic block designed for siRNA, mRNA, pDNA and protein cargo delivery. Upon cellular internalization, the pH-responsive ELP micelles disassembled, exposing cationic endosomal escape peptides (EEPs) that disrupted endosomal membranes and facilitated cargo release into the cytosol (Eweje et al., 2025).

ELP’s T_t_ can also be made pH-responsive when the guest residues are ionizable. Examples include ELPs containing basic residues such as R (arginine, pK_a_ ≈ 12), K (lysine, pK_a_ ≈ 10.4), or H (histidine, pK_a_ ≈ 6.0), and ELPs containing acidic residues such as D (aspartic acid, pK_a_ ≈ 3.9) and E (glutamic acid, pK_a_ ≈ 4.3). These ELPs can undergo a sharp, isothermal phase transition triggered by a small change in pH, enabling the design of pH-responsive ELP micelles that maintain nanoparticles structures at physiological pH but dissemble into monomers under acidic conditions, such as those found in the tumor environments, to trigger drug release. MacKay et al. studied two group of pH-responsive ELPs, including an acidic ELP with X = V: I: E = 1: 3 : 1 and a basic ELP with X = V: H: G: A = 1: 2: 1: 1 (Mackay et al., 2010). Their studies demonstrated that these charged ELPs undergo phase transitions at the pH corresponding to their isoelectric point, where their net charge is neutralized. The specific pH of this isothermal phase transition depends on the ELP’s sequence composition, chain length, concentration, and temperature. For ELPs containing 120 pentameric repeats, the acidic variant (pK_a_ = 5.29) showed an increase in T_t_ as pH rose from 3.8 to 7.4 (Figure 2C), whereas the basic variant (pK_a_ = 6.22) exhibited a decrease in T_t_ as pH increased from 5.5 to 8.0 (Figure 2D). These effects were more pronounced under low-pH conditions. Similar to pH-nonresponsive ELPs, pH-responsive ELPs displayed lower T_t_ at higher polymer concentrations or with longer chain length. A mathematical model was later developed to predict T_t_ of pH-responsive ELPs as a function of ELP length, concentration, and solution pH. When combined with cationic endosomal escape peptides (EEPs), pH-responsive ELP micelles can promote lysosomal escape of encapsulated cargos following cellular internalization (Eweje et al., 2025). In this design, Eweje et al. (2025) recombinantly fused EEPs to the hydrophobic terminus of the ELP_BC,_ which became sequestered within the micellar core upon self-assembly. Once the micelles encountered the acidic environment of lysosomes and disassembled, the EEPs were exposed, enabling them to disrupt lysosomal membranes and facilitate the release of micelle-loaded cargos into the cytosol (Figure 3).

ELPs are often recombinantly conjugated with receptor-binding proteins for targeted delivery or with therapeutic proteins for sustained delivery. The fusion partner’s physicochemical properties can shift ELP’s T_t_, a phenomenon termed the fusion ΔT_t_ effect, defined as ΔT_t_ = T_t_ (ELP–fusion) − T_t_ (ELP). Mackay and Christensen et al. compared the (VPGXG)_90_, where X = V: A: G = 5: 2: 3, with its fusions to six hydrophobic and six hydrophilic proteins (Mackay et al., 2010; Christensen et al., 2013). They found that ΔT_t_ correlated negatively with the fraction of hydrophobic surface area of the fusion protein, while solvent-exposed polar and charged residues counteracted this effect. Among all residue types, charged residues contributed most strongly to changes in ΔT_t_. Overall, the hydrophobicity and abundance of charged residues in the fusion protein determine whether the fusion depresses or elevates ELP’s T_t_.

### 2.2 ELP production and purification

ELPs have been recombinantly expressed in *E.coli*, yeast, and plant systems. Among them, *E. coli* is the most widely used host due to its high yield and low cost for ELP production. A comparison of ELP production in *E. coli* and yeast expression systems is summarized in Table 1 (Meyer and Chilkoti, 1999; Sallach et al., 2009). A major challenge in ELP production arises from the repetitive VPGXG coding sequences, which can lead to genetic instability and reduced expression levels. Additionally, certain hydrophobic or highly charged ELPs tend to be unstable or prone to degradation in *E.coli*. Plants, such as tomato, potato, and tobacco, have also been used to produce ELP fusion proteins, but these systems typically require substantially longer production timelines. Comprehensive reviews are available elsewhere (Floss et al., 2010).

**TABLE 1:** Comparison of ELP production by *E. coli* and yeast.

​	*E. coli*	Yeast
Host	*BL21(DE3)*	*Pichia pastoris*
Culture time	1–2 days	5–6 days
Yield	50–200 mg per 1 L culture	1–5 mg per 1 L culture
Advantages	• High yield • Cost-effective • Noncanonical amino acid incorporation	• Post-translational modification • Avoid endotoxin contamination
Limitations	• Lack post-translational modification • Endotoxin contamination	• Low yield • Limited noncanonical amino acid incorporation

**FIGURE 4:**
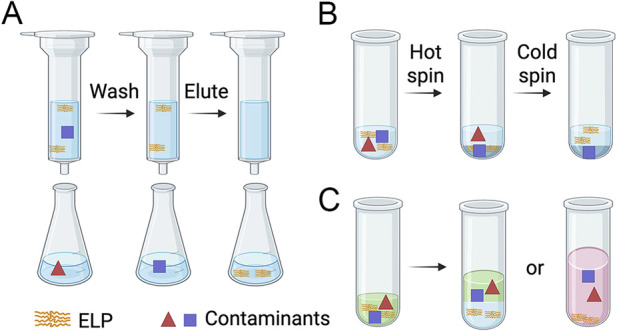
Schematic illustration of three ELP purification methods. **(A)** IMAC. His-tagged ELP binds to nickel-coated resins after loading samples. After washing away contaminants, ELP is eluted with imidazole. **(B)** ITC. In the hot spin, salt and heat induce ELP aggregation, allowing separation from soluble contaminants by centrifugation. In the cold spin, ELP pellets are dissolved in ice-cold buffer and re-centrifuged to remove insoluble contaminants. Dark blue indicates pellets, and light blue indicates supernatants. **(C)** Organic solvent extraction. Organic solvent is used to extract ELP from cell lysates or whole cells. ELP is then recovered either by back-extraction into an aqueous phase or by acetonitrile precipitation (light green: organic solvent for protein extraction; light blue: aqueous phase; light red: a mixture of organic solvent and acetonitrile).

ELPs can be purified by three major methods: immobilized metal affinity chromatography (IMAC), inverse transition cycling (ITC), and organic solvent extraction. A comparison of these methods is summarized in Table 2. In IMAC, ELPs are recombinantly fused with a His tag at either the C- or N-terminus, enabling selective binding to the metal ions such as nickel immobilized on a resin, separating His-tagged ELPs from non-tagged proteins and other contaminants (Haas et al., 2022) (Chae and Shin, 2025). Bound ELPs are eluted with imidazole (Figure 4A). After elution, dialysis or spin desalting is typically required to exchange buffer and remove imidazole and trace metal contaminants. IMAC can provide high-purity ELPs at relatively high cost. It is a well-established and reliable purification method widely used in recombinant protein purification. ITC, in contrast, purifies ELPs by using their thermo-responsive phase transition behaviors (Hassouneh et al., 2010). Each ITC consists of a hot spin and a cold spin (Figure 4B). In the hot spin, elevated temperature (typically 60 °C–70 °C) together with kosmotropic salts, such as NaCl and (NH_4_)_2_SO_4_, induce ELP coacervation. Centrifugation at 40 °C–50 °C separates the insoluble ELP aggregates from soluble impurities. In the subsequent cold spin, these ELP pellets are dissolved in ice-cold buffer and centrifugation at 4 °C separates the soluble ELPs from insoluble impurities. Typically, three to five ITCs yield ELPs with >95% purity. Additional cycles can increase the purity but may also reduce the overall recovery. Because ITC is nonchromatographic, it is simpler, more cost-effective, and readily scalable for industrial applications compared to IMAC. In addition to IMAC and ITC, ELPs can be extracted from whole *E.coli* cells or cell lysates using organic solvent extraction with pure solvents or solvent mixtures, followed by aqueous back-extraction or acetonitrile precipitation (Figure 4C) (Verheul et al., 2018; Sweet et al., 2021). This approach is faster than IMAC and ITC and is particularly advantageous for hydrophobic ELPs or those with poorly defined phase transition behaviors. Prior studies have shown that both small and large ELP-fusion proteins can be purified within 30 min using this method, yielding products with low endotoxin and DNA contaminants. Combining organic extraction with a single ITC cleanup step can further produce high-purify ELPs. However, organic solvents must be screened and optimized to maximize ELP extraction, and the efficiency of this approach for extracting diblock ELPs has not been studied.

**TABLE 2:** Comparison of three ELP purification methods.

​	IMAC	ITC	Organic extraction
Materials needed	His-tag, Nickel resin, imidazole	Salt, water bath	Organic solvent
Time	2–3 days	2–3 days	0.5–3 h
Advantages	• Highest ELP purify • Useful for ELP with low-yield or with very high or low T_t_ • One-step purification with His tag	• Nonchromatographic • Simple and cost-effective • Scalable • Avoid His-tag • Avoid organic solvent • Avoid imidazole	• Fast purification • Nonchromatographic • Avoid high salt • Useful for purifying hydrophobic ELPs
Limitations	• High cost • Need buffer exchange after purification • Imidazole is potentially hazardous • Includes a His-tag in the ELP	• Not applicable to very hydrophilic or very hydrophobic ELPs • Not applicable to ELPs without clear phase transition • High salt	• Organic solvent is hazardous • Organic solvent may denature some ELP fusion proteins • Require optimization of solvent type and mixture for maximum purification ability • Efficiency to purify ELP_BC_ has not been validated

### 2.3 Amphiphilic diblock ELP micelles

**FIGURE 5:**
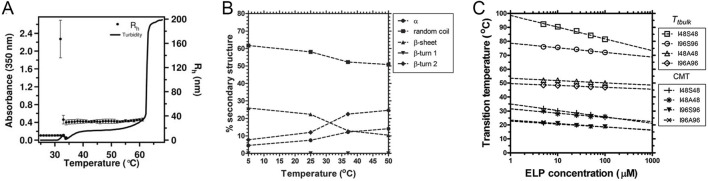
Unimer-micelle-aggregate transitions of ELP_BC_. **(A)** Turbidity (OD_350nm_) and hydrodynamic radius (R_h_) profiles of thioredoxin (Trx)-ELP_BC_ fusion at 25 μM in PBS. The ELP_BC_ had the sequence (VPGXG)_96_(VPGVG)_90_, where X = V: A: G = 1: 8: 7. DLS data showed the change in R_h_ with increasing temperature. The turbidity profile correlated with the change in R_h_. The unimer (R_h_ ≈ 7 nm) self-assembled into micelles (R_h_ ≈ 31 nm) at 35 °C, followed by micelle aggregation near 62 °C (Hassouneh et al., 2012a). **(B)** Changes in the percent secondary structure of the ELP diblock copolymer I48S48, which had the sequence of (VPGIG)_48_(VPGSG)_48_ (Janib et al., 2014). **(C)** Concentration-temperature phase diagrams of for ELP diblock copolymers I48S48, I48A48, I96S96, and I96A96 (Janib et al., 2014).

Rationally designed ELP_BC_, composed of a hydrophobic segment with T_t_ < 37 °C and a hydrophilic segment with T_t_ > 37 °C, can self-assemble into spherical or rod-like micelles at the physiological temperature. The T_t_ of the hydrophobic block defines the critical micelle temperature (CMT) while the T_t_ of the hydrophilic block corresponds to the bulk phase transition temperature (T_t,bulk_ or T_aggregation_), above which ELP_BC_ forms insoluble aggregates. The micelle is maintained between CMT and T_t,bulk_. Temperature-dependent turbidity or dynamic light scattering (DLS) measurements can well distinguish unimer-to-micelle transitions and micelle-to-aggregate transitions (Figure 5A) (Hassouneh et al., 2012a). Hydrophobic and hydrophilic ELP segments that have been used to construct ELP_BC_ micelles are summarized in Table 3.

**TABLE 3:** ELP segments used for micelle construction.

Hydrophobic ELP segments	Hydrophilic ELP segments
(VPGIG)_n_	(VPGSG)_n_
(VPGVG)_n_	(VPGAG)_n_
(VPGXG)_n_, X = W: V = 1: 4	(VPGXG)_n_, X = V: A: G = 1: 8: 7 or other ratios
(VPGFG)_n_	(VPGXG)_n_, X = G: A = 1: 1 or other ratios

Hassouneh and Dreher et al. demonstrated that ELP_BC_ form weak micelles consisting of dense hydrophobic cores and unstretched coronas, distinct from the tightly packed micelles formed by synthetic block copolymers. A mathematical model was developed to predict CMT, hydrodynamic radius (R_h_), and aggregation number (the number of ELP_BC_ molecules per micelle) of systems such as (VPGXG)_m_(VPGVG)_n_, where X = V: A: G = 1: 8: 7, m = 64 or 96, and n = 60, 90, or 120 (Hassouneh et al., 2015; Dreher et al., 2008). These micellar characteristics were found to depend on both the lengths of the hydrophobic and hydrophilic segments and the hydrophobicity of the guest residue. Specifically, increasing the length of the hydrophobic segment led to a lower CMT, larger R_h_, and higher aggregation number, while increasing the length of the hydrophilic segment resulted in an elevated T_t_,_bulk_, larger R_h_, and higher aggregation number, but had minimal influence on CMT. Additionally, R_h_ increased with rising temperature (Hassouneh et al., 2015; Dreher et al., 2008). Janib et al. later developed complementary mathematical models to predict CMT and T_t_,_bulk_ as functions of segment lengths and ELP concentrations after studying two sets of ELP_BC_ systems, including (VPGIG)_n_ (VPGSG)_n_, where n = 18, 24, 36, 48, or 96, and (VPGIG)_n_ (VPGAG)_n_, where n = 48 or 96 (Janib et al., 2014). Their finding revealed that stable ELP_BC_ micelle formation requires a minimum molecular weight. For example, in the (VPGIG)_n_(VPGSG)_n_ systems, only a single bulk phase transition was observed when n = 18, whereas unstable intermediate nanostructures formed at n = 24 or 48. Moreover, micelle formation was accompanied by a secondary-structure conversion in the hydrophobic segment from random coil and β-sheet conformations to type-II β-turns (Figure 5B). The effect of hydrophobic segment length on CMT was consistent across both ELP_BC_ systems; however, T_t,bulk_ of (VPGAG)_n_ displayed little length dependence, whereas a marked increase in T_t,bulk_ was observed for (VPGSG)_n_ (Figure 5C). Overall, these mathematical models—derived from systematic comparisons of ELP_BC_ with varying segment lengths and guest residues—provide a quantitative framework and guiding principles for the rational design of ELP-based micellar nanoparticles.

Micelles enable multivalent presentation of ligands on their surface, thereby enhancing ligands’ binding avidity to target receptors. Hassouneh et al. used (VPGXG)_96_(VPGVG)_90_ micelles, where X = V: A: G = 1: 8: 7, to present a fibronectin type III domain (Fn3) that specifically binds to α_v_β_3_ integrin. Fn3-decorated 60 nm ELP_BC_ micelles showed ∼2-fold increase in cellular uptake compared to unmodified micelles in the α_v_β_3_-overexpressing K562 human leukemia cell line (Hassouneh et al., 2012a). MacEwan et al. further designed (VPGVG)_40_(VPGXG)_60_, where X = A: G = 1: 1, which exhibits a CMT between 37 °C and 42 °C (Macewan and Chilkoti, 2014a). Their hydrophilic ends were recombinantly fused with cell penetrating peptides (CPPs). This ELP_BC_ enabled precisely controlling cellular uptake between “OFF” and “ON” by changing the environmental temperatures. At 37 °C, CPP-decorated ELP_BC_ unimers failed to enter cells efficiently due to lacking multivalent interaction between CPPs and their receptors. Under mild hyperthermia such as 42 °C, ELP_BC_ self-assembled into 40 nm micelles, facilitating multivalent binding and substantially enhancing cellular internalization. A 5–10-fold increase in cellular uptake at 42 °C compared with 37 °C was observed following 1 h incubation with CPP-decorated ELP_BC_. Such thermo-responsive ELP_BC_ micelles hold promise for hyperthermia-assisted cancer therapy, where localized heat exposure enhances the uptake of ELP micelle-delivered therapeutic drugs by tumor cells.

Other stimulus-responsive ELP_BC_ micelles have also been developed. Abdelghani et al. designed (VPGX_1_G)_60_(VPGX_2_G)_60_, where X_1_ = I: H = 1: 4 and X_2_ = A: G = 3: 2 (Abdelghani et al., 2021). This ELP_BC_ undergoes a reversible unimer-to-micelle transition in response to metal ions and acidic pH, due to the properties of the block containing H. The [I_1_H_4_]-based block is hydrophobic and aggregates at physiological pH but becomes hydrophilic in acidic environments due to the H residue protonation. Additionally, histidine residues can also chelate metal ions. Increasing Zn^2+^ concentration decreased micelle size and improved their stability at 37 °C. Interestingly, when this histidine-containing ELP_BC_ was mixed with another ELP_BC_ possessing identical hydrophilic segments but a (VPGIG)_60_ hydrophobic block, mixed micelles formed above the T_t_ of (VPGIG)_60_, exhibiting pH-independent assembly–disassembly behavior. Such mixed micelles have potential as multifunctional nanocarriers for co-delivering oligonucleotides and hydrophobic drugs—the protonated H residues bind nucleic acids electrostatically, while the hydrophobic micellar core encapsulates water-insoluble small molecules.

**FIGURE 6:**
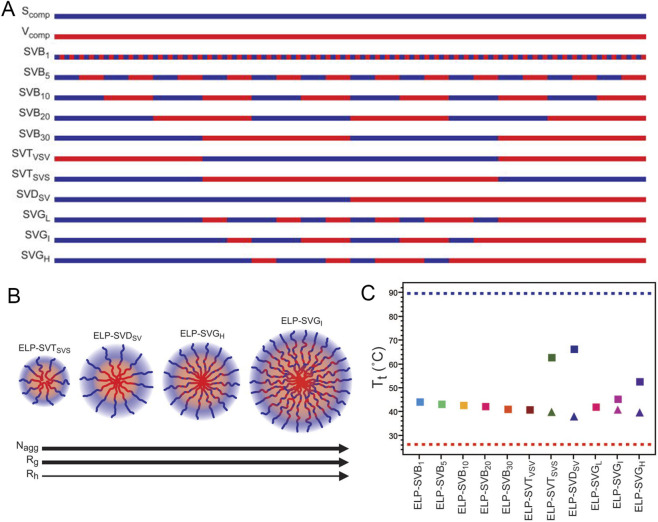
ELP’s architecture influences their self-assembly into micelles. **(A)** Design of ELPs with various block architecture. Each ELP was composed of 60 repeats of hydrophilic VPGSG (blue) and 60 repeats of hydrophobic VPGVG (red). Varying the organization of pentapeptides along the polypeptide chain resulted in ELPs with precisely defined architectures, including ELPs with alternating blocks of increasing block size, as well as triblock, diblock, and gradient ELPs. **(B)** Schematic illustration showing how ELP block copolymer architecture influences trends in R_h_, R_g_, and aggregation number (N_agg_). **(C)** Trends in T_t_ (squares) and CMT (triangles), dependent on ELP block copolymer architecture at concentrations of 100 μM. Increasing block size led to a decrease in unimer-to-aggregate phase transition T_t_, a trend that also included the unimer-to-micelle CMT when self-assembly behavior emerged. In contrast, the micelle-to-aggregate T_t_ increased with increasing hydrophobic gradients for self-assembled ELPs. SVS denotes a hydrophobic “V” block capped by two hydrophilic “S” blocks while VSV denotes a “S” block capped by two “V” blocks. L, I, and H indicate low, intermediate, and high gradients, respectively. (Macewan et al., 2017).

In addition to diblock ELPs, triblock and mutli-block ELPs can also self-assemble into micelles. MacEwan et al. studied the phase transition behavior and micelle formation capability of a series of precisely defined ELP_BC_ with varying architectures (Macewan et al., 2017). Each construct was composed of 60 repeats of VPGSG and 60 repeats of VPGVG, but the sequence arrangement of these repeats differed among the variants. In the group of ELPs with evenly distributed hydrophobic and hydrophilic blocks, polymers containing four or more alternating blocks (ELP-SVB_1_ thorough ELP-SVB_30_) were unable to form micelles at any of the tested temperatures, and their T_t_ slightly decreased with increasing block size (Figure 6). Interestingly, triblock ELPs with hydrophilic segments at the termini (ELP-SVT_svs_) can form micelles, whereas those with hydrophobic segments at the termini (ELP-SVT_vsv_) cannot. In the group of ELP with a gradient distribution of hydrophobic blocks, micelle formation was not observed at low hydrophobic gradients (ELP-SVG_L_). In this study, for ELP_BC_ variants capable of forming micelles, both the micelle size and aggregation number varied despite having identical overall compositions. This work well demonstrated that block architecture critically governs the micellization ability, size, and morphology of self-assembled ELPs.

Overall, ELP micelles are highly tunable platforms for drug delivery. Both stimulus-responsive or non-responsive ELP micelles can be rationally designed by modulating the guest residue composition, chain length, and block architectures. By exploiting the intrinsic physicochemical properties of ELPs and environmental triggers such as temperature or pH, researchers can direct ELPs to self-assemble into micelles for enhanced cellular uptake through multivalent ligand presentation or to disassemble selectively at diseased sites—such as tumors—to achieve controlled drug release.

### 2.4 ELP-lipid/polymer/peptide/drug hybrid micelles

Attachment of hydrophilic ELPs to hydrophobic moieties, such as lipids or short hydrophobic peptides (e.g., (YG)_n_ or (FGG)_n_), creates amphiphiles that can self-assemble into micelles. Researchers have conjugated hydrophilic ELPs, such as those with X = A: V = 9:1 or X = A: V = 2: 8 or X = V: M = 2: 1, or X = V, with myristoyl chains, cholesterol, or fatty acids *via* post-translational modification or chemical reaction, and characterized their phase transition behavior and potential applications (Luginbuhl et al., 2017; Mozhdehi et al., 2018; Mozhdehi et al., 2019; Zhang et al., 2023; Zhang et al., 2024). These ELP-lipid hybrids self-assemble into spherical or rod-like micelles or fibers or droplets independent of temperature. Lipid chain type and length influence the critical micelle concentration (CMC) and micelle size, whereas the ELP length primarily affects micelle size. Compared to ELP_BC_ micelles, ELP-lipid micelles exhibit greater structural stability due to the high hydrophobicity of lipids, resulting in more efficient and durable encapsulation of hydrophobic small-molecule drugs.

Hydrophilic ELPs have been recombinantly fused to silk polypeptide or resilin-like polypeptide (RLPs) to form micelle or micelle-like nanoparticles in which ELPs form the micellar corona. Silk blocks composed of (GAGAGS)_n_ mimic the repetitive motifs of silkworm fibroin heavy chain and naturally assemble into tightly packed β-sheet structures (Huang et al., 2015). Xia et al. compared a group of silk-ELP (SELP) copolymers with sequences of [(GXGVP)_8_ (GAGAGS)_n_]_14–9_, where X = V: Y = 7: 1, n = 1, 2 or 4 (Xia et al., 2011; Xia et al., 2014). These SELPs were designated SE8Y, S2E8Y, and S4E8Y, respectively. SE8Y and S2E8Y exhibited two-step thermal transitions, including the first transition at ∼40 °C, corresponding to silk block aggregation into hydrophobic β-sheets, and the second transition at ∼60 °C, corresponding to ELP coacervation. SELPs form 200–250 nm micelles within 40 °C–60 °C. In contrast, S4E8Y exhibited only the silk-associated transition at ∼95 °C and no subsequent ELP transition, likely due to its high silk-to-elastin ratio, which masked ELP behavior.

**FIGURE 7:**
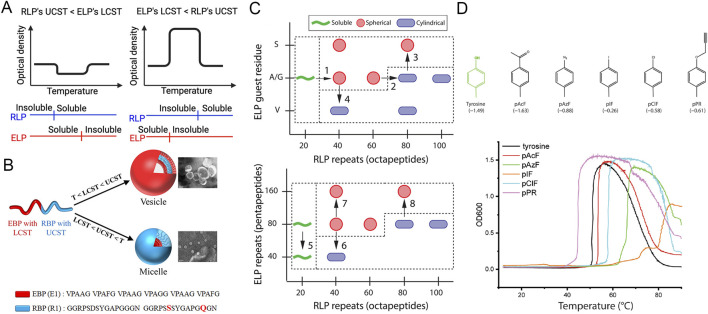
RLP-ELP self-assemble into micelles. **(A)** Schematic illustration displaying two types of ELP-RLP constructs. If RLP’s UCST is lower than ELP’s LCST, micelles or other types of nanoparticles are formed when T < UCST or T > LCST. If ELP’s LCST is lower than RLP’s UCST, micelles or other types of nanoparticles are formed when T < LCST or T > UCST. Insoluble polypeptides form the micellar core while soluble polypeptide form the micellar corona. **(B)** RLP-ELP (termed RBP-EBP) with the sequence E_6_R_n_ (n ≥ 6) self-assembled into vesicles when T < LCST < UCST and formed micelles with LCST < UCST < T (Basheer et al., 2021). **(C)** Quasi-phase diagrams illustrating trends in RLP-ELP self-assembly into spherical or cylindrical micelles. RLP-ELP had the sequence (QYPSDGRG)_n_ (XGVPG)_80_, where X = A/G or S or V (Weitzhandler et al., 2017). **(D)** Chemical structures of the aromatic side chains of tyrosine and unnatural amino acids incorporated in RLP-ELP genes (logD values are indicated in parentheses) and representative turbidity profiles as a function of temperature for RLP_40_ (TAG×12)-ELP_60_, which had the sequence [(RGDSPYSGRGDSPYSGRGDSP*SG)_3_RGDSPYSG]_4_-(VPGGGVPGAG)_30_. * denotes the TAG codon, which were conjugated with unnatural amino acids (Azulay et al., 2024).

While ELPs typically exhibit a LCST transition—soluble below T_t_ and insoluble above it, RLPs exhibit an upper critical solution temperature (UCST) transition—insoluble below T_t_ and soluble above it (Balu et al., 2021; Quiroz and Chilkoti, 2015). Rationally designed LCST-UCST diblock copolymers show unique temperature-dependent self-assembly, in which the hydrophobicity of each block reverses as temperature increases, producing micelles with interchanging core-corona compositions across distinct temperature ranges. Weitzhandler et al. recombinantly synthesized RLP-ELP containing RLP blocks (QYPSDGRG)_n_ and ELP blocks (VPGXG)_n_, where X = A: G = 1 : 1 (Weitzhandler et al., 2017). In this construct, the RLP segments underwent UCST transitions before the LCST transitions of the ELP blocks. They self-assemble into micelles consisting of RLP core and ELP corona at temperatures below RLP’s UCST, and they dissembled into unimers between RLP’s UCST and ELP’s LCST. Theoretically, they will form micelles or vesicles with a hydrophilic RLP corona and a hydrophobic ELP core when the temperature is above ELP’s LCST (Figure 7A, left panel). This study also showed that when the RLP repeat number increased from 40–60 to 80–100, micelle morphology shifted from spherical to cylindrical. Substituting the ELP segments with more hydrophilic (VPGSG)_n_ or more hydrophobic (VPGVG)_n_ also influenced micelle size and morphology (Figure 7C).

Basheer et al. constructed RLP-ELP copolymers, in which RLP’s UCST was greater than ELP’s LCST (Basheer et al., 2021). The RLP block (“R”) had the sequence (GGRPSDSYGAPGGGN GGRPSSSYGAPGQGN), while the ELP block (“E”) had the sequence (VPAAG VPAFG VPAAG VPAGG VPAAG VPAFG). When the temperature was below ELP’s LCST, the construct E_12_R_24_, consisting of 12 “E” blocks and 24 “R” blocks, formed 68 nm micelles with an RLP core and an ELP corona. In contrast, E_6_R_n_ (n = 6, 12, or 24) self-assembled into larger 272–702 nm vesicles, characterized by an RLP hydrophobic layer sandwiched between inner and outer hydrophilic ELP layers. When the temperature was greater than RLP’s UCST, these constructs reassembled into 48–118 nm reverse micelles with an ELP core and an RLP corona (Figure 7B). This reversible transition between micelles, unimers, and reverse micelles illustrates a switchable dual-thermo-responsive system with potential for drug delivery applications (Figure 7A, right panel).

Azulay et al. further expanded the functionality of RLP-ELP copolymers by incorporating aromatic unnatural amino acids (uAA) into the RLP block (Azulay et al., 2024). Their constructs had the sequences of (GRGDSPYS)_n_(VPGXG)_60_, where n = 20, 40, or 60, and X = G: A = 1: 1. In these genes, TAG codons were strategically distributed throughout the RLP sequence, replacing ∼30% of the tyrosine residues with uAAs. Five different aromatic uAA, including P-acetyl-L-phenylalanine (pAcF), P-azido-L-phenylalanine (pAzF), 4-Iodo-L-phenylalanine (pIF), 4-Chloro-L-phenylalanine (pCIF), and 4-propargyloxy-L-phenylalanine (pPR), were incorporated into RLP-ELP individually. These RLP-ELP copolymers formed 50–80 nm micelles with an RLP core and an ELP corona at temperatures below RLP’s UCST. Incorporation of uAAs modulated RLP hydrophobicity and π–π stacking interactions, thereby shifting the micelle formation temperature (Figure 7D). Moreover, the RLP/ELP block length ratio and uAA hydrophobicity determined micellar morphology. RLP-ELP copolymers with longer or more aromatic RLPs preferentially form cylindrical micelles.

Cylindrical micelles have also been observed in systems in which hydrophilic (VPGXG)_n_, where X = A: G = 1: 1, or (VPGAG)_n_ were recombinantly fused to short aromatic peptides, such as (XGG)_8_, where X = F or W, or (XG)_8_, where X = Y or F (McDaniel et al., 2014; Saha et al., 2022). These ELP-based cylindrical micelles, comprehensively reviewed elsewhere (Saha et al., 2020), can present more surface ligands than spherical micelles, thereby enhancing multivalent avidity toward cell-surface receptors and promoting greater cellular uptake of micelles and their cargos (Dzuricky et al., 2019).

Conversely, hydrophobic ELPs conjugated with hydrophilic synthetic polymers, such as poly (ethylene glycol) (PEG) or poly (acrylic acid) (PAA), can form amphiphilic copolymers that self-assemble into micelles above the ELP’s transition temperature (T_t_). The design, synthesis, and biomedical applications of these ELP–polymer hybrid micelles have been reviewed in detail elsewhere (Van Eldijk et al., 2014; Paik et al., 2015).

### 2.5 ELP-drug micelle-like nanoparticles

Hydrophobic or hydrophilic small molecule drugs can be covalently conjugated to the C (cysteine) residues of hydrophilic ELP that are recombinantly fused to a short peptide including C, such as (GGC)_n_. Micellar self-assembly can then be triggered by the hydrophobicity of conjugated drugs or by a hydrophobic polymer segment within the segment. Conjugation of drugs within ELP nanoparticles can enhance their loading amount (for hydrophobic drugs), circulation half-life, tumor accumulation, and intracellular uptake, while also protecting drugs from enzymatic degradation. McDaniel et al. systematically examined how the hydrophobicity of conjugated compounds influences micelle formation using an ELP construct of SKGPG-(XGVPG)_160_-WPC(GGC)_7_, where X = V: A: G = 1: 8: 7 (McDaniel et al., 2013b). They found that attachment of compounds with logD ≤1.5 did not trigger ELP self-assembly, whereas compounds with logD >1.5 induced micelle formation. The aggregation number (number of ELP conjugates per nanoparticle) increased with the hydrophobicity of the conjugated compound. An ELP-drug conjugate incorporating the hydrophobic chemotherapeutic doxorubicin *via* an acid-labile hydrazone linker self-assembled into sub-100 nm spherical micelles that demonstrated superior tumor suppression compared with free drug in C26 murine colon cancer preclinical models (Andrew Mackay et al., 2009). Bhattacharyya et al. further conjugated a group of hydrophilic small molecule drugs to the cysteine residues of (AGVPG)_160_(YG)_6_(CGG)_8_ and successfully obtained 40–140 nm self-assembled cylindrical micelles, driven by the hydrophobic (YG)_6_ segment (Bhattacharyya et al., 2017a). In these constructs, hydrophilic drugs likely localized at the core-corona interface or within the hydrophilic ELP corona. An ELP conjugated with hydrophilic gemcitabine self-assembled into ∼60 nm rod-like micelles and exhibited enhanced tumor inhibition relative to free gemcitabine in HCT-116 human colon cancer preclinical models (Bhattacharyya et al., 2017a).

Hydrophilic therapeutic proteins can be recombinantly fused to ELP_BC_ and presented on the micelle surface after ELP_BC_ self-assembly. Alternatively, they can also be fused to enable formation of micelles with a hydrophobic ELP core and a protein corona. Huang et al. constructed RGD-TRAIL-ELP, in which the ELP has the sequence (VPGXG)_40_, where X = V: H = 1: 4. This conjugate self-assembled into 190 nm micelle-like nanoparticles at 37 °C (Table 4) (Huang et al., 2017). Here, RGD serves as an integrin-binding motif, and TRAIL (tumor necrosis factor-related apoptosis-inducing ligand, ∼20 kDa) is a pro-apoptotic therapeutic protein. These RGD-TRAIL-ELP nanoparticles exhibited prolonged systemic circulation, enhanced tumor accumulation, and improved antitumor efficacy compared with soluble RGD-TRAIL in COLO-205 human colon cancer preclinical models. Similarly, Koria, Yeboah, and Kang et al. conjugated an ELP, consisting of 40 repeats of VPGVG and 2 repeats of (VPGVG)_2_(VPGCG)(VPGVG)_2_, to different therapeutic proteins, including KGF, SDF1a, and vRAGE, for wound healing applications. These ELP-protein fusions form ∼500 nm nanoparticles above ELP’s T_t_ (Koria et al., 2011; Yeboah et al., 2016; Kang et al., 2021). Sarangthem et al. recombinantly fused AP1 (RKRLDRN), an IL-4 receptor (IL-4R)-binding peptide that provides tumor-homing ability, and (KLAKLAK)_2_, a pro-apoptotic peptide known as KLAK, to the ELP (Sarangthem et al., 2021). The resulting fusion, AP1-ELP-KLAK, had the sequence [AP1-(VPGVG)_12_]_6_-KLAK and self-assembled into 110–130 nm nanoparticles at the physiological temperature. These targeted ELP micelles exhibited significantly enhanced cytotoxicity toward IL-4R-overexpressing U87MG and D54 human glioblastoma cells, demonstrating superior antitumor efficacy compared with untargeted ELP-KLAK nanoparticles *in vitro* and *in vivo*.

**TABLE 4:** Summary of targeted ELP micelles used for drug delivery in cancer therapy.

​	ELP sequence	Target receptor	Drug	Micelle characterization	*In vitro* performance	*In vivo* performance	Disease models	References
RGD-ELP_BC_/FKBP-ELP_BC_/rapamycin mixed micelles	G(VPGIG)_48_(VPGSG)_48_Y-GRGDGG mixed with FKBP-G(VPGSG)_48_(VPGIG)_48_Y	integrin	Rapamycin	∼50 nm	• Comparable IC_50_ to untargeted micelles	• Higher tumor accumulation, and better efficacy than untargeted micelles	MDA-MB-468 human breast cancer in nude mice	Dhandhukia et al. (2017)
RGD-TRAIL-ELP micelles	RGD-TRAIL-(VPGXG)_40_, X = V: H = 1 : 4	integrin	TRAIL	∼190 nm	• Lower EC_50_ than RGD-TRAIL	• Half-life: ∼3.83 h vs. 0.85 (RGD-TRAIL) • Higher tumor accumulation, and better efficacy than RGD-TRAIL.	COLO-205 human colon cancer in nude mice	Huang et al. (2017)
AP1-ELP-KLAK micelles	[AP1-(VPGVG)_12_]_6_-(KLAKLAK)_2_	IL-4R	KLAK	110–130 nm	• Stronger apoptosis induction in U87MG and D54 cells than untargeted ELP-KLAK micelles	• Greater tumor apoptosis than ELP-KLAK nanoparticles after intravenous injection	D54 human glioblastoma in nude mice	Sarangthem et al. (2021)
Tat-AP1-ELP/siRNA nanoparticles	Tat-A_1_E_28_: Tat-AP1-[(VPGVG)_5_(VPGFG)_2_-(VPGVG)_3_(VPGGG)_3_(VPGAG)]_2_ Tat-A_4_V_48_: Tat-[AP1-(VPGVG)_12_]_4_	IL-4R	siRNA against luciferase gene	534 nm (siRNA/Tat-A_1_E_28_) 247 nm (siRNA/Tat-A_4_E_48_)	• Tat-AP1-ELP/siRNA showed higher *in vitro* transfection efficiency in IL-4R^+^ cells than Tat-ELP/siRNA and ELP/siRNA	• Tat-AP1-ELP/siRNA exhibited enhanced tumor accumulation and greater gene silencing efficacy in tumor cells than free siRNA, Tat-ELP/siRNA, and ELP/siRNA	4T1 murine breast cancer in BALB/c mice	Yi et al. (2020)
Tat-AP1-ELP/miRNA nanoparticles	Tat-A86: Tat-(AP1-V_5_)_16_-[(V_3_G_3_A_1_)_3_V_7_]_3_ A86: (AP1-V_5_)_16_-[(V_3_G_3_A_1_)_3_V_7_]_3_ Tat-E60: Tat-(V_3_G_3_A_1_)_16_(V_7_)_5_	IL-4R	miRNA-34a	145.5 nm (Tat-A86/miRNA) 31.64 nm (A86/miRNA) 126.9 nm (Tat-E60/miRNA)	• Tat-A86/miRNA induced higher apoptosis in LLC cell and organoid than the other two formulations	• Tat-A86/siRNA exhibited greater tumor suppression than free miRNA and the other two formulations	LLC murine lung cancer in C57BL/6 mice	Hong et al. (2024)

IC_50_ or EC_50_: half maximal inhibitory or effective concentration.

ELPs fused with cationic DNA-binding sequences rich in R and K residues can neutralize and compact nucleic acids through electrostatic interactions, forming self-assembled ELP/nucleic acid nanocomplexes suitable for DNA or RNA delivery. Nouri et al. fused different (VPGXG)_8_ sequences, where X = A: G = 3: 1 or S: G = 3: 1 or E: G = 3: 1 or K: G = 3: 1, with RH_3_, a cationic peptide with the sequence of (RRVRRSHRRRHT)_3_. RH_3_ can condense plasmid DNA (pDNA). The resulting ELP/pDNA complexes formed 180–200 nm nanoparticles, whereas RH_3_/pDNA and RH_3_-PEG_3500_/pDNA formed ∼50 nm nanoparticles. In these assemblies, hydrophilic ELPs constituted the corona, protecting the encapsulated pDNA in the core from enzymatic degradation. *In vivo* studies demonstrated that these ELP/pDNA complexes were non-immunogenic, eliciting no significant IgG responses, while RH_3_-PEG_3500_/pDNA nanocomplexes induced mild IgG responses (Nouri et al., 2015). Similarly, Chen et al. fused a short peptide K_8_ to (VPGXG)_60_, where X = V: A: G = 5: 2: 3, and found that their complex with pDNA formed nanoparticles ranging from 32.4 to 115.5 nm, varied with N/P ratios. Increasing the N/P ratios reduced the nanoparticle size. Remarkably, the transfection efficiency of ELP/pDNA nanoparticles at an N/P ratio of 1 was comparable to that of poly (ethyleneimine) (PEI)/pDNA complexes at an N/P ratio of 10 in MCF-7 human breast cancer cells (Chen et al., 2008). A major advantage of using ELPs for nucleic acid delivery is their genetic modularity. Peptide-based homing ligands or cell-penetrating peptides can be recombinantly fused to ELPs and displayed on the nanoparticle surface to achieve targeted delivery and enhanced cellular uptake. For example, Sarangthem and Hong et al. developed ∼145 nm ELP/miRNA-34a nanoparticles using an ELP construct named Tat-A86, composed of TAT-[AP1-(VPGVG)_5_]_16_-[(VPGXG)_21_(VPGVG)_7_]_3_, where X = V: G: A = 3: 3: 1 (Table 4) (Sarangthem et al., 2020; Hong et al., 2024). miRNA-34a directly targets 3′-UTRs of multiple oncogenic mRNAs to suppress tumor progression. In this construct, TAT (YGRKKRRQRRR) is a cell penetrating peptide that can condense miRNA. IL-4R-binding AP1 peptides further enhanced cellular uptake of ELP nanoparticles. Compared to ELP/miRNA nanoparticles lacking AP1 or TAT, Tat-A86/miRNA nanoparticles exhibited markedly higher intracellular delivery efficiency and superior antitumor efficacy in LLC murine lung cancer preclinical models (Hong et al., 2024).

### 2.6 Drug loading to ELP micelles

Protein and peptide drugs can be recombinantly fused to ELP, achieving a 100% conjugation ratio (Hassouneh et al., 2012a). With optimized plasmid design, these proteins can be precisely inserted at the N- or C-terminus of ELP and additional peptide linkers or targeting ligands can be incorporated at designated positions (McDaniel et al., 2010). This genetic modularity represents a unique advantage of ELP nanoparticles over synthetic polymer-based carriers for peptide and protein drug delivery.

Small-molecule drugs, in contrast, are typically chemically conjugated to ELPs *via* short peptide handles that contain reactive functional groups such as thiols (–SH) in cysteine residues. Incorporation of unnatural amino acids further expands the available chemical functionalities for drug attachment (Costa et al., 2019). Besides, stimuli-responsive chemical linkers—cleavable by enzymes, acidic pH, or reactive oxygen species—can be introduced to engineer controlled and site-specific drug release from ELP nanoparticles (Su et al., 2021).

**FIGURE 8:**
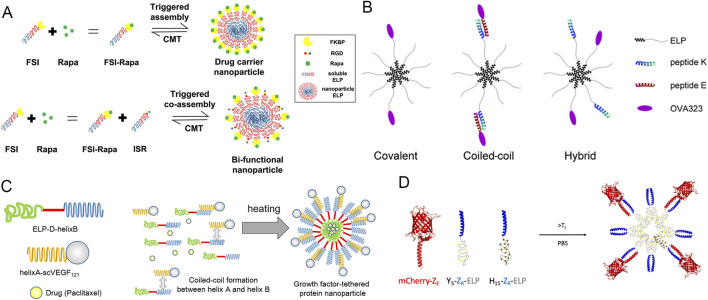
Schematic illustration showing drug loading to the ELP micelle surface *via* noncovalent interactions. **(A)** Rapamycin (Rapa) binds to FKBP proteins recombinantly fused to ELP_BC_ (FSI) (Dhandhukia et al., 2017). **(B)** OVA-peptide E and ELP-peptide K self-assemble into micelles displaying OVA on the surface. Peptide K interacts with peptide E through coiled-coil formation, anchoring OVA to the micelle (Van Strien et al., 2024). **(C)** ELP-D-helixB and helixA-scVEGF self-assemble into ELP micelles displaying VEGF on the surface. HelixA binds to helixB via coiled-coil interaction, anchoring scVEGF to the micelle (Assal et al., 2015). **(D)** mCherry-Z_E_, Y_5_-Z_R_-ELP, and H_15_-Z_R_-ELP co-assemble into ELP nanoparticles displaying mCherry on the surface. Z_E_ binds to Z_R_ through coiled-coil pairing, anchoring mCherry to the nanoparticle surface (Gray et al., 2025).

Drugs can also be loaded onto ELP micelles through noncovalent interactions. MacKay group fused diblock ELPs with FK506-binding protein 12 (FKBP), a 12 kDa protein that binds hydrophobic rapamycin, to create rapamycin-loaded ELP micelles. These formulations showed enhanced therapeutic efficacy in preclinical models of cancer and autoimmune disease (Figure 8A) (Shah et al., 2013; Dhandhukia et al., 2017; Shi et al., 2013). Similarly, Chen group fused ELP to small synthetic IgG-binding domains, such as the Z or ZZ domain, to form tight noncovalent complexes with IgG molecules and then use these Z- or ZZ-decorated ELP nanoparticles for IgG purification. Z domain is a three-helix bundle engineered from Staphylococcal protein A (SpA), while ZZ domain is a tandem repeat of two Z domains (Madan et al., 2013; Swartz et al., 2018). Kros group developed ∼57 nm spherical coiled-coil micelles by mixing the fusion of amphiphilic ELP with peptide “K” ((KIAALKE)_n_) and the conjugate of peptide “E” ((EIAALEK)_n_) with the model antigen OVA323. Peptides “K” and “E” form a heterodimeric coiled-coil complex, enabling multivalent display of OVA323 on the ELP micelles (Figure 8B) (Van Strien et al., 2024). The coiled-coil interaction between K and E peptides enabled multivalent antigen display on micelle surfaces. Compared with chemically conjugated counterparts, these self-assembled micelles exhibited greater antigen uptake by dendritic cells, promoting enhanced maturation and CD4^+^ T cell proliferation. Similarly, Kobatake group employed complementary α-helical coiled-coil peptides, helix A (ACID-p1, AQLEKELQALEKENAQLEWELQALEKELAQ) and helix B (BASE-p1, AQLKKKLQALKKKNAQLKWKLQALKKKLAQ), to tether growth factors onto ELP micelles for targeting cancer cells overexpressing growth factor receptors, achieving potent antitumor effects *in vitro* (Figure 8C) (O'Shea et al., 1993; Assal et al., 2015). Champion group utilized noncovalent interactions between arginine-rich leucine zippers (Z_R_) and glutamine-rich leucine zippers (Z_E_) to anchor hydrophilic globular proteins on ELP micelle surfaces (Figure 8D). They found that modulating ELP hydrophobicity and chain length allows precise control of nanoparticle and vesicle morphology (Li et al., 2023; Gray et al., 2025; Gray et al., 2024).

In addition to these strategies, enzymatic and biorthogonal conjugation systems have been developed for attaching peptide and protein cargos to ELPs. Examples include SpyTag/SpyCatcher, which forms an irreversible isopeptide bond between a 13-amino-acid peptide (SpyTag) and its 15 kDa protein partner (SpyCatcher), and enzyme-mediated ligation catalyzed by sortase A (SrtA) or SnoopLigase (Swartz and Chen, 2018a; Swartz and Chen, 2018b). Comprehensive discussions of these covalent bioconjugation strategies can be found in recent reviews (Park et al., 2023).

### 2.7 Targeted ELP micelles

When peptide or protein homing ligands are recombinantly fused to the hydrophilic end of amphiphilic ELP_BC_, they are expected to display on the micelle surface following self-assembly, thereby conferring targeting capability. Hydrophilic peptides, such as short affinity peptide (RGD, NGR) and cell penetrating peptides (CPPs), can be successfully presented on the micelle surface without requiring additional peptide linkers between the ELP_BC_ and the ligand (Dreher et al., 2008; Weinberger et al., 2017; Macewan and Chilkoti, 2013; MacEwan and Chilkoti, 2012; Simnick et al., 2011; Simnick et al., 2010; Walker et al., 2012; Walter et al., 2024). However, to properly display slightly hydrophobic homing peptides, a rationally designed hydrophilic peptide linker must be inserted between the ELP and the ligand. Without such a linker, peptide ligands tend to be buried within the micellar core or corona during self-assembly, losing accessibility to target receptors.

**FIGURE 9:**
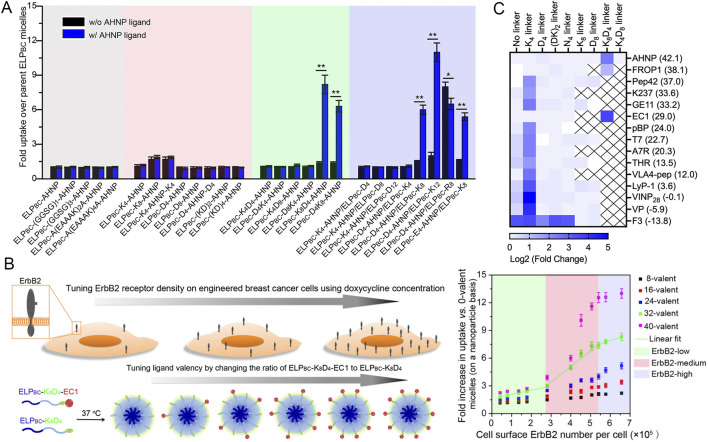
ELP_BC_ micelles modified with optimized hydrophilic peptide linkers correctly display hydrophobic homing ligands on the micelle surface. **(A)** Cellular uptake of ELP_BC_–linker–AHNP micelles and controls in ErbB2-overexpressing SK-BR-3 breast cancer cells (Wang et al., 2017a). **(B)** Cellular uptake of ELP_BC_-K_8_D_4_–EC1 micelles by MCF-7/Tet-On/ZsGreen-ErbB2 cells at varying receptor and ligand densities. ErbB2 expression on the cell surface was tuned by doxycycline. Ligand valency on ELP_BC_ micelles was tuned by mixing EC1-modified with unmodified ELP_BC_-K_8_D_4_ at defined ratios (Wang J. et al., 2020). AHNP and EC1 are hydrophobic peptides that binds to ErbB2. **(C)** Fold change in uptake of ELP_BC_–linker–ligand micelles normalized to their corresponding ELP_BC_–linker controls in receptor-overexpressing cells. For each ligand, hydrophilic linkers differing in hydrophilicity and charge were tested. A group of homing peptide ligands with varying hydrophobicity was tested (numbers in parentheses denote hydrophobicity index). Heat map redrawn from the referenced data (Wang et al., 2019). ELP_BC_ used in these studies had the sequence of (VPGXG)_40_(VPGSG)_40_, where X = W: V = 1: 4.

Wang et al. systematically evaluated a series of hydrophilic linkers with varying hydrophilicity and charge in the construct (VPGXG)_40_(VPGSG)_40_ micelles, where X = W: V = 1: 4, to optimize the display of two hydrophobic ErbB2 (HER2)-targeting peptides, AHNP (FCDGFYACYMDV; hydrophobicity index (HI) = 42.1) and EC1 (WTGWCLNPEESTWGFCTGSF; HI = 29). They found that inclusion of a K_8_D_4_ linker provided sufficient hydrophilicity for proper display of these ligands on the ELP_BC_ micelle surface. The resulting ELP-K_8_D_4_-AHNP and ELP-K_8_D_4_-EC1 micelles exhibited markedly higher cellular uptake in ErbB2/HER2^+^ cells compared to micelles lacking the hydrophilic linker (Figure 9A). Similar results were obtained with mixed micelles containing ELP-K_8_ and ELP-D_4_, which produced comparable hydrophilicity. Interestingly, surface charge also influenced ligand presentation. While ELP–K_8_D_4_-AHNP micelles (ζ = −2.0 ± 0.6 mV) efficiently promoted ErbB2-dependent uptake, ELP–K_4_D_8_-AHNP micelles (ζ = −8.2 ± 0.4 mV) with similar hydrophilicity failed to induce receptor-mediated internalization (Wang et al., 2017a). Using these optimized targeted ELP micelles, Wang et al. further fine-tuned the ligand valency on the micelle surface by mixing unmodified ELP-K_8_D_4_ with ELP-K_8_D_4_-EC1 at varying ratios. The resulting mixed micelles maintained consistent size, shape, and aggregation behavior, indicating that incorporation of short peptides did not alter micellar architecture. Comprehensive studies using engineered breast cancer cells with tunable ErbB2 expression levels revealed how ligand density and receptor density jointly influence multivalent ligand-receptor interactions and the ensuing receptor-mediated cellular uptake (Figure 9B) (Wang J. et al., 2020). In another study, Wang et al. further examined 15 peptide ligands (HI = −13.8–42.1) using eight different linkers (K_4_, D_4_, (DK)_2_, N_4_, K_8_, D_8_, K_8_D_4_, D_4_K_8_). They concluded that, for (VPGXG)_40_(VPGSG)_40_ micelles, where X = W: V = 1: 4, a minimum of a hydrophilic cationic K_4_ linker is required for optimal display of ligands with moderate hydrophobicity (HI = −6–24), while a more hydrophilic K_8_D_4_ linker is necessary for highly hydrophobic ligands (HI > 24) (Figure 9C) (Wang et al., 2019). In contrast, hydrophilic long peptides and proteins can be directly displayed on ELP micelles without additional linkers (Hassouneh et al., 2012a; Wang et al., 2019). Collectively, these studies underscore the critical role of micellar surface hydrophilicity and charge in achieving effective ligand display and targeting. Similar design principles have also been validated in synthetic polymer micelle systems (Stefanick et al., 2013a; Stefanick et al., 2013b).

## 3 Applications of ELP micelle drug delivery systems in different diseases

ELP micelles offer multiple advantages as platforms for drug delivery. First, biodegradable ELPs exhibit excellent biocompatibility and low immunogenicity *in vivo*, likely because these bioinspired elastin-like materials closely resemble endogenous elastin. Additionally, ELPs are degradable *in vivo*, and their breakdown products are natural amino acids, thereby reducing the risk of toxicity (Shah et al., 2012). *In vivo* studies of various ELP formulations (e.g., microparticles, depots, and hydrogels) across multiple animal models (e.g., mice, rats, and rabbits) have demonstrated that ELPs exhibit no systemic toxicity and minimal intrinsic immunogenicity (Rincón et al., 2006; Cho et al., 2016; Zhang et al., 2015; Camal Ruggieri et al., 2024; Waller et al., 2021). Interestingly, unlike synthetic “stealth” polymers such as PEG, which can elicit anti-PEG IgG production, ELPs have been shown not to trigger detectable antibody responses, underscoring their non-immunogenic nature (Ong et al., 2006; Urry et al., 1991b). Although ELP–antigen fusions can markedly enhance antigen-specific antibody production by increasing antigen size and multivalency, the resulting antibodies do not cross-react with ELP, and ELP alone does not elicit detectable antibody responses (Ingrole et al., 2014). Early clinical trials such as NCT01523067 and NCT01873885 suggested that ELPs can be well tolerated in humans and usually do not trigger significant immune responses, even though no ELP carrier is FDA-approved yet (Paulis et al., 2015). The biocompatibility of ELPs has been reviewed elsewhere (Frandsen and Ghandehari, 2012; Despanie et al., 2016; Macewan and Chilkoti, 2014b; Hassouneh et al., 2012b).

Second, ELP micelles can prolong systemic circulation of small-molecule drugs following intravenous administration. Renal clearance is strongly size-dependent, as the glomerular filtration membrane in the kidney has a pore size of approximately 6 nm, which effectively prevents filtration of nanoparticles larger than this threshold. ELP micelles typically range from 30 to 500 nm in diameter and thus evade rapid renal elimination, whereas small molecules, peptides, and short proteins are rapidly cleared, resulting in limited circulation times (Du et al., 2018). Increasing ELP micelle size prolongs circulation and reduces renal accumulation (Kuna et al., 2018). Circulation half-life is also influenced by surface charge and interactions with plasma proteins. The circulation of ELP micelles can be further extended through fusion with albumin-binding proteins or by employing zwitterionic ELPs (Yousefpour et al., 2018; Banskota et al., 2019; Banskota et al., 2020).

Third, ELP micelles enhance tumor accumulation of therapeutic cargos *via* the enhanced permeability and retention (EPR) effect, while minimizing off-target accumulation in major organs. Owing to the leaky vasculature characteristic of tumor tissues, nanoparticles readily extravasate into the tumor microenvironment but are inefficiently cleared. In contrast, small molecules freely diffuse across both normal and abnormal vasculature, leading to nonselective distribution (Jasim et al., 2017). The preferential retention of ELP micelles at tumor sites improves therapeutic efficacy while reducing systemic toxicity. Moreover, their biodistribution can be tuned by altering the length, architecture, or surface charge of ELP micelles (Sarangthem et al., 2020).

Fourth, ELP micelles can encapsulate or conjugate a broad spectrum of therapeutics, including small molecules, nucleic acids, peptides, and proteins, and can enable co-delivery of multiple agents. Drugs are typically localized within micellar core or corona, protected from enzymatic degradation. Additionally, stimuli-responsive ELP micelles can be designed to respond to pH, hypoxia, temperature, or enzymatic cues, enabling controlled drug release at disease sites. Their combination with hyperthermia therapy can further enhance tumor-specific uptake of therapeutics.

In addition to the advantages shared with polymer-based micelle drug delivery system, ELPs offer unique benefits. As protein-based materials, ELPs are well suited for them as a promising delivery system for peptide therapeutics because drugs can be recombinantly fused to ELPs, ensuring precise stoichiometry and eliminating the need for organic solvents during conjugation and purification. Targeted ELP micelles can be also achieved by recombinantly fusing peptide ligands (e.g., RGD, AP1, or antibodies) to the ELP’s hydrophilic terminus, ensuring their display on the micelle surface. Moreover, ligand density can be precisely tuned by mixing ligand-conjugated and unmodified ELP_BC_ at defined ratios (Wang J. et al., 2020). Additionally, degradable ELPs may offer improved *in vivo* safety compared with synthetic polymers, as their degradation products are natural amino acids.

ELP micelles have been successfully used for the delivery of a wide range of therapeutic agents and have demonstrated strong antitumor efficacy in preclinical models. In the following sections, we summarize the applications of ELP micelles and micelle-like nanoparticles for cancer therapy and vaccine development, emphasizing studies that have evaluated their *in vivo* performance. For cancer therapy, chemotherapeutic and immunotherapeutic agents are typically administered *via* intravenous injection, where ELP micelles have been shown to extend drug circulation, enhance tumor accumulation, and reduce systemic toxicity compared to free drugs. Incorporating targeting ligands further augments tumor accumulation and therapeutic efficacy (Tables 4, 5). In vaccine applications, which are generally delivered subcutaneously or intramuscularly, ELP micelles have been utilized to deliver antigens. Vaccination aims to activate antigen-presenting cells (APCs)—notably dendritic cells and macrophages—to elicit cellular (T-cell) and humoral (antibody) immune responses. Effective immunization can protect hosts from pathogen infection or tumor challenge (Table 6). Adjuvant is an important component within vaccines, which are used to boost and shape the immune response (e.g., Th1 or Th2 responses). Traditional adjuvants include aluminum salts, Freund’s adjuvant, and Toll-like receptor (TLR) agonists such as monophosphoryl lipid A and CpG, etc. Recent studies demonstrate that ELP micelle-delivered antigens, even without additional adjuvants, can efficiently enhance APC activation and antigen presentation, thereby improving vaccination efficacy.

**TABLE 5:** Summary of untargeted ELP micelles used for drug delivery in cancer therapy.

​	ELP sequence	Drug	Micelle characterization	*In vitro* performance	*In vivo* performance	Disease models	References
ELP-doxorubicin micelles	SKGPG-(XGVPG)_160_-WPC(GGC)_7_, X = V: A: G = 1: 8 : 7	Doxorubicin	∼40 nm, spherical, ∼3 μM CAC	• Faster drug release at pH 5.3 vs. 7.4 • Higher IC_50_ than free drug	• Half-life: 9.3 ± 2.1 h vs. ∼5 min (free drug) • ↑ Tumor and ↓ cardiac accumulation • Lower toxicity and better efficacy than free drug	C26 murine colon cancer in BALB/c mice	Andrew Mackay et al. (2009)
ELP-paclitaxel micelles	SKGPG-(XGVPG)_160_-WPC(GGC)_7_, X = V: A: G = 1: 8: 7	Paclitaxel	∼60 nm, spherical, ∼1 μM CAC	• Faster drug release at pH 5.3 vs. pH 7.4 • Slightly higher IC_50_ than free drug	• Half-life: 8.4 ± 1.9 h vs. 3.8 ± 0.7 h (Abraxane) • ↑ Tumor accumulation • Lower toxicity and better efficacy than free drug and Abraxane	MDA-MB-231 human breast cancer and PC3 human prostate cancer in nude mice	Bhattacharyya et al. (2015)
ELP-niclosamide micelles	SKGPG-(XGVPG)_160_-WPC(GGC)_7_, X = V: A: G = 1: 8: 7	Niclosamide	Sub-100 nm, cylindrical, ∼3 μM CAC	• Comparable IC_50_ to free drug	• Half-life: 4.2 h • Better efficacy than free drug	HCT-116 human colon cancer in nude mice	Bhattacharyya et al. (2017b)
ELP-salinomycin micelles	(GAGVPG)_70_-(CG_8_)_8_	Salinomycin	∼110 nm	• Comparable IC_50_ to free drug	• Half-life: 4.45 h vs. 0.86 h (free drug) • Better efficacy than free drug	4T1 murine breast cancer in BALB/c mice	(Zhao et al., 2014) (Zhao et al., 2016)
FKBP-ELP_BC_/rapamycin micelles	FKBP-G(VPGSG)_48_(VPGIG)_48_Y	Rapamycin	∼64 nm	• Extended terminal drug release compared to FKBR-free ELP micelles encapsulating rapamycin solely in the core • Comparable IC_50_ to free drug	• Lower toxicity, better efficacy than free drug	MDA-MB-468 human breast cancer in nude mice	Shi et al. (2013)
ELP chemically conjugated with Pt (IV) or chelating Pt (II)	(AGVPG)_160_-(YG)_6_-(XGG)_8_, X = C for Pt conjugation and X = D for Pt chelation	Platinum	∼130 nm, cylindrical, 0.3–0.6 μM CAC	• ATBP- ATBP-Pt (IV) released drug slower than ATBP-Pt (II) at pH 5 • Higher IC_50_ than free drug	• Half-life: ∼9 h vs. ∼0.2 h (free drug) • Lower toxicity and better efficacy than free drug • ATBP-Pt (IV) outperformed ATBP-Pt (II) at equitoxic dose	FaDu human head and neck cancer in nude mice	Saha et al. (2022)
α-FLT3 scFv-ELP nanoparticles	α-FLT3-G(VPGAG)_192_Y	α-FLT3 scFv	∼77 nm	• Stronger binding to FLT3^+^ cells than α-FLT3 scFv	• Half-life: 14.7 h vs. 2.34 h (α-FLT3 scFv) • Comparable efficacy to midostaurin	MOLM-13 human leukemia model in NSG mice	Park et al. (2020)
α-CD99 scFv-ELP nanoparticles	α-CD99-G(VPGAG)_192_Y	α-CD99 scFv	∼94 nm	• Specific binding to CD99^+^ cells	• Half-life: ∼16 h • Reduce leukemia burden and extend animal survival	MOLM-13 human leukemia model in NSG mice	Vaikari et al. (2020)
IFNα-ELP_BC_ micelles	IFNα-(VPGAG)_48_(VPGIG)_48_	IFNα	∼50 nm	• Weaker binding to IFNαR^+^ cells than IFNα • Higher IC_50_ than free IFNα	• Half-life: 54.7 h vs. 9.6 h (IFNα-ELP monomer) vs. 0.44 h (IFNα) vs. 39.0 h (PEGylated IFNα) • Better efficacy than IFNα-ELP monomer and free IFNα	OVCAR-3 human ovarian tumor in nude mice	Wang et al. (2020b)

IC_50_: half maximal inhibitory concentration. A higher IC_50_ value indicates lower cytotoxicity.

**TABLE 6:** Summary of ELP micelles used for vaccine delivery.

​	ELP sequence	Antigen	Micelle characterization	*In vivo* performance	Animal strain (doses)	References
Antigen-ELP_BC_ nanoparticles	MESLLP-Antigen-[(VPGVG)_2_(VPGEG)(VPGVG)_2_]_10_-[VPGIG]_60_V	*Mycobacterium tuberculosis*-derived peptide	∼55 nm ∼4 µM CAC −12.1 mV in PBS	• Induced IL-1β and IL-5 secretion and generated antigen-specific IgM and IgG after immunization • Free antigen and control ELP_BC_ micelles failed to elicit antigen-specific immune responses	BALB/c mice (three doses)	García-Arévalo et al. (2013)
pOVA-ELP_BC_ micelles	G(GAGVPG)_70_-G(GVLPGVG)_28_-pOVA	OVA-derived pOVA	∼82 nm	• Elicited stronger antigen-specific CD8^+^ T cell responses than full-length OVA or OVA_1_ peptide	C57BL/6 mice (two doses)	Cho et al. (2016)
H100-ELP_BC_-pOVA	H100-[(GAGVPG)_35_(GVLPGVG)_16_]_2_-pOVA	pOVA	T_t_ > 37 °C	• Both H100-ELP/ELP-pOVA mixture and H100-ELP-pOVA triggered stronger antigen-specific CD8^+^ T cell responses than ELP-pOVA combined with Freund’s adjuvant	C57BL/6 mice (two doses)	Dong et al. (2017)
ELP_BC_/ELP_BC_-Bet v1 mixed micelles	M(GVPGI)_48_(GVPGS)_48_-Bet v1	The major birch pollen allergen Bet v1	∼45 nm 0.22 µM CMC −6.8 mV in PBS	• Induced earlier and higher production of IgG1 and IgG2a compared with aluminum salt-adjuvated Bet v1 formulations	BALB/c mice (three doses)	Van Strien et al. (2022)
OVA-Z_E_/pZ_R_-ELP vesicles	pZ_R_(VPGXG)_25_, X = V: F = 4: 1, F residues were partially replaced by pAzF during protein expression	Full-length OVA	∼175 nm −12.8 mV	• Generated greater IgG1 and IgG2a levels and enhanced antigen-specific CD4^+^ T and CD8^+^ T cell responses compared to soluble OVA-Z_E_	BALB/c (two doses)	Li et al. (2024); Li et al. (2023)

### 3.1 ELP micelles delivering small molecule chemotherapeutic drugs

In 2009, MacKay and Chen et al. developed ELP-doxorubicin micelles where small molecule chemotherapeutic drug doxorubicin was conjugated to the cysteine residues of (GGC)_8_ appended to the hydrophilic (VPGXG)_160_, where X = V: A: G = 1: 8: 7, *via* pH-labile linkers (Table 5) (Andrew Mackay et al., 2009). Driven by the hydrophobicity of doxorubicin, ELP-doxorubicin self-assembled into ∼40 nm micelles with a critical aggregation concentration (CAC) of ∼3 μM ELP. The acid-sensitive linker enabled doxorubicin release at pH 5, a typical pH value at the tumor and endosomal environments, but remained stable at physiological pH 7.4. After cellular internalization, doxorubicin was released from acidic lysosomes, diffused into the cytosol, and subsequently entered the nucleus to intercalate DNA and induce cytotoxicity in tumor cells (Wang et al., 2017b). ELP-doxorubicin micelles enhanced the plasma half-life of doxorubicin from several minutes to 9.3 ± 2.1 h and increased the area under the concentration-time curve (AUC) from 4.7 μM-h to 716 ± 139 μM-h. Compared with free drugs, ELP-doxorubicin micelles exhibited 3.5-fold higher tumor accumulation and 2.6-fold lower cardiac accumulation at 24 h after intravenous injection, effectively mitigating cardiotoxicity. The maximum tolerated dose (MTD) was ∼4-fold higher for ELP-doxorubicin micelles, and antitumor efficacy was markedly superior compared to free doxorubicin. In C26 murine colon cancer preclinical models, a single intravenous dose of ELP-doxorubicin micelles at early cancer stages yielded a 90% survival rate, compared with only 10% in mice treated with free doxorubicin (Andrew Mackay et al., 2009). Enhanced therapeutic efficacy was also confirmed in 4T1 murine breast cancer preclinical models. A subsequent study revealed that ELP-doxorubicin micelles reprogrammed intratumoral myeloid cells towards a pro-inflammatory, anti-tumor phenotype, thereby activating CD8^+^ T cells to exert antitumor activity—a mechanism distinct from the direct cytotoxicity of free doxorubicin (Mastria et al., 2018).

In 2015, Bhattacharyya et al. covalently conjugated paclitaxel to the same hydrophilic ELP *via* acid-sensitive linkers, which self-assembled into ∼60 nm ELP-paclitaxel micelles (Table 5) (Bhattacharyya et al., 2015). Paclitaxel, a hydrophobic microtubule-stabilizing chemotherapeutic that arrests cell division, served as both the therapeutic cargo and hydrophobic driver for micelle formation. These ELP-paclitaxel nanoparticles exhibited superior antitumor efficacy compared to the FDA-approved formulation Abraxane® (∼130 nm paclitaxel–HSA complexes). In MDA-MB-231 human breast cancer models, a single intravenous dose yielded a mean tumor volume of 150 mm^3^ at 6 weeks, versus 216 mm^3^ for Abraxane and 535 mm^3^ for free paclitaxel. Similarly, in PC3 human prostate cancer models, mean tumor volume was 23 mm^3^ for ELP-paclitaxel micelles, compared with 216 mm^3^ for Abraxane and 549 mm^3^ for free drug at 30 days post-injection. The ability of ELP-drug nanoparticles to suppress tumor growth in immunodeficient mice suggested that the micelles enhance paclitaxel delivery efficiency and may engage innate immune mechanisms. In 2017, Bhattacharyya et al. conjugated hydrophobic niclosamide, an anthelmintic that inhibits Wnt/β-catenin signaling, to the same ELP platform to overcome drug’s poor solubility, low bioavailability, and rapid clearance (Table 5) (Bhattacharyya et al., 2017b). The resulting ELP-niclosamide conjugates self-assembled into sub-100 nm cylindrical micelles with a CAC of ∼3 μM and efficiently suppressed tumor growth in HCT-116 human colon cancer preclinical models.

In 2014, Zhao et al. applied a similar strategy by conjugating hydrophobic salinomycin—a selective cancer stem-cell toxin—to an immune-tolerant, hydrophilic ELP, forming stable drug-loaded micelles (Table 5) (Zhao et al., 2014; Zhao et al., 2016). Incorporation of dimethylhexylamine (DMHA) and α-tocopherol improved drug loading and release behavior. These micelles extended salinomycin’s plasma half-life from 0.86 h to 4.45 h and increased tumor accumulation ∼2.4-fold at 12 h post-injection. Consequently, micelle-delivered salinomycin suppressed tumor growth and eradicated cancer stem cells more efficiently than free drug in 4T1 murine breast cancer models. A follow-up study confirmed that combining salinomycin-ELP and paclitaxel-loaded ELP micelles achieved synergistic tumor suppression and prolonged survival compared to either monotherapy (Zhao et al., 2016).

In 2013, Shi et al. designed ELP_BC_-FKBP nanoparticles, in which FKBR, the cognate protein target of rapamycin, was fused to the hydrophilic end of (VPGSG)_48_(VPGIG)_48_ (Table 5) (Shi et al., 2013). The FKBP domain bound rapamycin noncovalently, while the hydrophobic core also physically encapsulated the drug (Figure 8A). Following drug loading, the nanoparticle diameter increased from ∼45 nm to ∼64 nm. Dialysis experiments revealed biphasic release kinetics. In the FKBP-ELP_BC_ rapamycin-loaded micelles, ∼70% of the drug was encapsulated within the hydrophobic core, while the remaining ∼30% was bound to FKBP in the hydrophilic corona. Drug released from the core displayed a fast release phase (t_1/2_ = 1.9 h) whereas those bound in the corona displayed a slow-release phase (t_1/2_ = 57.8 h). In contrast, FKBP-free rapamycin-loaded micelles, which encapsulated the drug solely within the hydrophobic core, showed a single-phase exponential decay (t_1/2_ = 2.2 h). This extended drug release profile of rapamycin-loaded FKBP-ELP_BC_ micelles translated into improved antitumor efficacy in MDA-MB-468 human breast cancer models, increasing survival from 0% to 90% compared with free rapamycin. This study highlighted the advantages of affinity-based, noncovalent drug loading in prolonging release and enhancing antitumor activity. In 2017, Dhandhukia et al. further developed ∼50 nm mixed micelles by co-assembling RGD-ELP_BC_ and FKBP-ELP_BC_ at a 1: 1 molar ratio, enabling simultaneous tumor targeting and rapamycin binding (Table 4) (Dhandhukia et al., 2017). The RGD peptide provided integrin-mediated homing, while FKBP retained high affinity to rapamycin. Both targeted and untargeted micelles exhibited similar *in vitro* cytotoxicity and pharmacokinetics, but targeted micelles achieved ∼2-fold higher tumor accumulation and significantly improved therapeutic efficacy in MDA-MB-468 human breast cancer preclinical models, without added toxicity (Peddi et al., 2020). These findings underscore the potential of targeted ELP nanoparticles to deliver drugs specifically to tumors while maintaining favorable pharmacokinetics.

In 2022, Saha et al. reported two ELP-based micelles for delivering hydrophilic platinum (Pt) chemotherapeutic drugs, including ELP-(YG)_6_-(CGG)_8_ chemically conjugated with Pt (IV) and ELP-(YG)_6_-(DGG)_8_ chelating Pt (II) (Table 5) (Saha et al., 2022). The ELP backbone used in this study was the hydrophilic (AGVPG)_160_, while the hydrophobic (YG)_6_ segment drove self-assembly of the asymmetric triblock conjugates into ∼130 nm cylindrical micelles. The terminal cysteine or aspartate residues enabled Pt coordination or covalent conjugation (Bhattacharyya et al., 2017a). These two Pt-loaded ELP micelles, termed asymmetric triblock polypeptide ATBP-Pt (IV) and ATBP-Pt (II), exhibited CAC of 0.3–0.6 μM. At pH 5, ATBP-Pt (II) released free drug much faster than ATBP-Pt (IV) because the protonation of carboxylate groups in ATBP-Pt (II) disrupted the Pt (II) coordination bond, whereas the thioester bond in ATBP-Pt (IV) was more stable and cleaved at a slower rate. Under reducing conditions such as ascorbate buffer, thioester cleavage was markedly accelerated, triggering rapid drug release from ATBP-Pt (IV) micelles. *In vivo*, both Pt-loaded micelles achieved AUC values of 1004–1119 μM-h and half-lives of ∼9 h at a 5 mg/kg dose, greatly exceeding those of free cisplatin (AUC = 27.8 ± 1.8 μM-h, t_1/2_ = 0.2 ± 0.02 h). In FaDu head and neck cancer preclinical models, both formulations exhibited lower systemic toxicity and enhanced antitumor therapeutic efficacy than free drug. Notably, ATBP-Pt (IV) outperformed ATBP-Pt (II) at equitoxic dose, likely reflecting its greater *in vivo* stability and more favorable drug-release profile.

### 3.2 ELP micelles delivering peptide and protein drugs

#### 3.2.1 Cancer therapy

In 2017, Huang et al. designed an RGD-TRAIL-ELP trimer, in which the hydrophobic ELP sequence was (VPGXG)_40_, where X = V: H = 1: 4 (Table 4) (Huang et al., 2017). Tumor necrosis factor-related apoptosis-inducing ligand (TRAIL) is a type II membrane protein that binds to death receptors to induce apoptosis in tumor cells. In this study, the soluble extracellular domain of TRAIL (∼20 kDa) was used. The resulting RGD-TRAIL-ELP self-assembled into ∼190 nm nanoparticles at the physiological temperature and exhibited a ∼4.5-fold longer half-life and ∼2.5-fold higher tumor accumulation than RGD-TRAIL after intravenous injection. In COLO-205 human colon cancer preclinical models, a single intraperitoneal injection of RGD-TRAIL-ELP induced nearly complete tumor regression and outperformed RGD-TRAIL.

In 2020, Park and Vaikari et al. developed α-FLT3 scFv-ELP micelles, termed α-FLT3-A192, in which the hydrophilic ELP sequence was (VPGAG)_192_ (Table 5) (Park et al., 2020). The single-chain variable fragment (scFv) comprises the variable regions of the antibody’s heavy and light chains and contains complementarity-determining residues that contribute to antigen binding. Fusion of the α-FLT3 scFv to the hydrophilic ELP induced self-assembly into micelles, likely with a scFv core and an ELP corona, lowering the ELP’s T_t_ from ∼60 °C to ∼42 °C. Although the scFv contributes to micellar core formation, some scFv molecules may still remain exposed on the surface of these dynamic nanoparticles. Therefore, α-FLT3 scFv-ELP micelles enhanced binding to FLT3^+^ cancer cells compared to the free α-FLT3 scFv. A similar increase in binding avidity was observed with α-PD-1 scFv conjugated to the hydrophilic terminus of an ELP_BC_, which localized on the micellar surface after self-assembly (Zhao et al., 2017). *In vivo*, α-FLT3 scFv-ELP nanoparticles exhibited a half-life of 14.7 h, compared with 2.34 h for the free α-FLT3 scFv. In MOLM-13 leukemia preclinical models, α-FLT3 scFv-ELP nanoparticles efficiently reduced leukemia burden in both peripheral blood and bone marrow, achieving efficacy comparable to midostaurin, an FDA-approved FLT3 inhibitor for acute myeloid leukemia (AML). These nanoparticles also moderately extended animal survival. Vaikari and Park et al. further used the same ELP to develop α-CD99 scFv-ELP rod-like micelles for leukemia treatment (Table 5) (Vaikari et al., 2020). Like FLT3, CD99 is overexpressed in AML and represents a promising therapeutic target. The α-CD99 scFv-ELP micelles demonstrated a ∼16 h half-life, specific binding to CD99^+^ cells *in vitro*, and potent antitumor efficacy in leukemia preclinical models.

In 2020, Wang et al. designed IFNα-ELP_BC_ micelles by recombinantly fusing interferon alpha (IFNα, ∼19kD) cytokines to the hydrophilic end of (VPGAG)_48_(VPGIG)_48_ (Table 5) (Wang Z. et al., 2020). The resulting 50 nm spherical micelles did not outperform the conjugate of IFNα with a single hydrophilic ELP (IFNα-ELP) in binding to cancer cells overexpressing IFNα receptors (IFNαR). Both formulations—IFNα-ELP_BC_ micelles and IFNα-ELP monomers—exhibited comparable anti-proliferative activity in IFNαR^+^ cells, and both were less potent than free IFNα. It’s likely that IFNα was not properly displayed on the micelle surface, limiting their access to IFNαR and compromising the multivalency effect. Despite the *in vitro* limitations, *in vivo* results demonstrated superior antitumor efficacy of IFNα-ELP_BC_ micelles compared to IFNα-ELP monomers and free IFNα in OVCAR-3 human ovarian cancer preclinical models. The improvement was attributed to their enhanced pharmacokinetic profile. IFNα-ELP_BC_ micelles exhibited a half-life of 54.7 h, markedly longer than that of free IFNα (0.44 h) and IFNα-ELP monomers (9.6 h) and even surpassing PEGylated IFNα (39.0 h).

In 2021, Sarangthem et al. designed AP1-ELP-KLAK nanoparticles that used AP1 homing ligands to specifically deliver pro-apoptotic KLAK peptides to IL-4R^+^ cells (Table 4) (Sarangthem et al., 2021). Six copies of the AP1 peptide were evenly inserted within the ELP backbone, conferring targeting ability to IL-4R^+^ tumor cells. Both *in vitro* and *in vivo* studies demonstrated that AP1-decorated ELP-KLAK micelles induced significantly greater apoptosis in IL-4R^+^ U87MG and D54 human glioblastoma cells compared to non-targeted controls. In D54 human glioblastoma preclinical models, intravenous administration of AP1-ELP-KLAK for eight consecutive days dramatically inhibited tumor growth.

#### 3.2.2 Vaccine development

In 2013, Garcia-Arevalo et al. recombinantly fused a peptide antigen derived from a major membrane protein of *Mycobacterium tuberculosis* to the hydrophilic end of an ELP_BC_ with the sequence of (VPGXG)_50_(VPGIG)_60_, where X = E: V = 1: 4 (Table 6) (García-Arévalo et al., 2013). Notably, no additional immunostimulatory adjuvants were included in the formulation. The antigen-ELP_BC_ self-assembled into ∼55 nm nanoparticles. *In vivo* immunization experiments were conducted by subcutaneously administering the antigen-ELP_BC_ nanoparticles, or free antigen, or control antigen-free ELP_BC_ nanoparticles at days 0, 14, and 28. Cytokine profiling and serum antibody analyses at multiple time points suggested that antigen-ELP_BC_ nanoparticles elicited a biphasic response. The initial phase corresponded to innate immune activation, characterized by secretion of chemotactic cytokines such as IL-1β, followed by a secondary adaptive immune phase marked by IL-5 (a Th2 cytokine) production and the generation of antigen-specific IgM and IgG antibodies. After immunization, naïve B cells first secrete IgM, and with T-cell help, they class-switch to IgG, producing higher-affinity, longer-lived antibodies. Consistent with this process, IgM peaked around day 7 after the first immunization with antigen-ELP_BC_ nanoparticles and gradually declined, while IgG levels steadily increased and peaked by day 35. In contrast, neither free antigen nor control ELP_BC_ nanoparticles elicited detectable humoral responses. This enhanced vaccination efficacy was attributed to the ability of ELP nanoparticles to enable multivalent antigen display and improve trafficking to the draining lymph nodes, which together promoted efficient dendritic cell uptake, antigen presentation, and subsequent activation of T and B cells, ultimately inducing antigen-specific adaptive immunity.

In 2016, Cho et al. developed a group of ELPs and demonstrated their low immunogenicity *in vivo*. With these ELPs, they engineered an ELP_BC_ with the sequence of (GAGVPG)_70_(GVLPGVG)_28_, and recombinantly fused a fragment of the model antigen ovalbumin (OVA) to its hydrophobic end (Table 6) (Cho et al., 2016). OVA, a 45 kD protein from egg while, is widely used in immunology as a model antigen because its two peptide fragments elicit well-defined T cell response. OVA_1_ (OVA_257-264_, SIINFEKL) can induce a strong CD8^+^ T cell response while OVA_2_ (OVA _323–339_, ISQAVHAAHAEINEAGR) can induce a strong CD4^+^ T cell response. In this study, the fused antigen—designated pOVA—contained two tandem copies of the OVA_1_ epitope (ESIINFEKLTESIINFEKLT). The resulting pOVA-ELP_BC_ conjugates self-assembled into ∼82 nm micelles, encapsulating pOVA in the micellar core. These micelles exhibited ∼1.3-fold and ∼1.6-fold higher efficiency in activating DC2.4 dendritic cells for OVA_1_ antigen presentation compared with full-length OVA and pOVA fused to a hydrophilic ELP, respectively. Consequently, pOVA-ELP_BC_ micelle-treated dendric cells can more efficiently activate CD8^+^ T cells in the co-culture assays. *In vivo*, mice immunized intramuscularly with two doses of pOVA-ELP_BC_ micelles on days 1 and 15 exhibited ∼1.5-fold higher levels of antigen-specific CD8^+^ T cells in the spleen compared with mice receiving full-length OVA or OVA_1_ peptides. In 2017, Dong et al. from the same group recombinantly fused pOVA and a self-adjuvanting peptide, H100, to a multi-block ELP with the sequence of [(GAGVPG)_35_(GVLPGVG)_16_]_2_ (Table 6) (Dong et al., 2017). Although this construct shared a similar composition with the previously described ELP_BC_, it did not form micelles at physiological temperatures. H100 is a fragment derived from high-mobility group box 1(HMGB1), known to mature and activate dendritic cells and thus induce antigen-specific CD8^+^ T cell response. Mice were immunized either with a mixture of H100–ELP and ELP-pOVA or with a bifunctional H100–ELP–pOVA construct, in which H100 and pOVA were fused to the two termini of ELP, respectively. ELISPOT experiments indicated that both vaccination strategies significantly increased the abundance of antigen-specific CD8^+^ T cells in the spleen compared with mice receiving pOVA–ELP plus Freund’s adjuvant, a conventional vaccine adjuvant. The study also showed that fusing H100 to either the N- or C-terminus of ELP monomers did not affect their ability to mature and activate dendritic cells *in vitro*.

In 2022, van Strien et al. fused Bet v1 antigen, a 17.6kD major birch pollen allergen, to an ELP_BC_, (GVPGI)_48_(GVPGS)_48_, and mixed it with antigen-free ELP_BC_ at a 1: 9 ratio to prepare a ∼45 nm mixed micelle (Table 6) (Van Strien et al., 2022). Mice immunized with ELP_BC_/ELP_BC_-Bet v1 mixed micelle produced IgG1 (Th2-biased antibody) and IgG2a (Th1-biased antibody) earlier and at higher levels than Bet v1 absorbed to aluminum salt, a commonly used adjuvant. It’s known that both antibody subclasses are important for vaccine-induced protection, with IgG2a providing stronger pathogen- and tumor-clearing activity. Serum cytokine profiling revealed that both formulations primarily induced Th2 cytokines, such as IL-5 and IL-13, but not Th1 and Th17 cytokines, such as IFNγ and IL-17A.

In 2024, Li et al. utilized an affinity binding approach to noncovalently conjugate OVA to ELP vesicles (Table 6) (Li et al., 2024; Jang et al., 2017). OVA was recombinantly fused to an acidic leucine zipper (Z_E_) and mixed with ELP fused to a basic leucine zipper (Z_R_). The interaction between OVA-Z_E_ and Z_R_-ELP, followed by ELP self-assembly, yielded ∼175 nm vesicles with multivalent OVA display on the surface (Figure 8D). To stabilize the vesicles, the unnatural amino acid pAzF was incorporated into Z_R_-ELP (pZ_R_-ELP) to allow photocrosslinking. Upon immunization, these OVA-Z_E_/pZ_R_-ELP vesicles elicited robust OVA-specific IgG1 and IgG2a antibody responses, whereas soluble OVA-Z_E_ failed to generate detectable antibodies. Consistently, OVA vesicles induced stronger antigen-specific CD4^+^ and CD8^+^ T-cell responses in mice than soluble controls, underscoring the immunostimulatory role of multivalent antigen display and nanoscale organization. Interestingly, pZ_R_-ELP alone triggered modest IgG1 production—contrary to prior findings that ELPs are non-immunogenic—likely resulting from crosslinking or/and the Z_R_ domain (Cho et al., 2016).

Collectively, these findings suggest that ELP micelles enhance immunization efficiency by extending the *in vivo* residence time of peptide antigens, and promoting antigen internalization through multivalent presentation or particle size effects.

### 3.3 ELP micelles delivering nucleic acid drugs

In 2020, Yi et al. developed two targeted ELP nanoparticles, Tat-A_1_E_28_ and Tat-A_4_V_48_, for delivering siRNA against the luciferase (Luc) gene (Table 4) (Yi et al., 2020). Small interfering RNA (siRNA) is a short, double-stranded RNA that silences gene expression through RNA interference. Tat-A_1_E_28_ had the sequence Tat-AP1-[(VPGVG)_5_(VPGFG)_2_-(VPGVG)_3_(VPGGG)_3_(VPGAG)]_2_ while Tat-A_4_V_48_ had the sequence Tat-[AP1-(VPGVG)_12_]_4._ Tat cell-penetrating peptides and IL-4R-binding AP1 peptides enhance uptake in IL-4R^+^ tumor cells while facilitating siRNA condensation through their cationic residues. The hydrophobic ELP segments promoted self-assembly into nanocomplexes ranging from 247 to 534 nm in diameter. These nanoparticles effectively protected siRNA from RNase degradation. The presence of Tat and AP1 significantly enhanced cellular uptake and gene-silencing efficiency in IL-4R^+^ MDA-MB-231 human breast cancer cells, compared to siRNA mixed with untargeted ELP (E_28_) or ELP fused with Tat (Tat-E_28_). The *in vitro* transfection efficiency of Tat-A_4_V_48_ was comparable to Lipofectamine, a commercial cationic polymer widely used for gene delivery. *In vivo*, intravenous administration of Tat-A_1_E_28_/siRNA and Tat-A_4_V_48_/siRNA nanocomplexes in 4T1/Luc murine breast cancer models resulted in 2-3-fold higher tumor accumulation and greater luciferase gene silencing compared to free siRNA, Tat-E_28_/siRNA nanoparticles, or E_28_/siRNA mixtures. Notably, the multivalent AP1 design in Tat-A_4_V_48_ conferred better targeting and gene silencing efficacy in both *in vitro* and *in vivo* settings compared to the monovalent Tat-A_1_E_28_ construct.

In 2024, Hong et al. applied a similar strategy to deliver miRNA-34a with Tat-AP1-ELP nanoparticles for cancer therapy (Table 4) (Hong et al., 2024). microRNA (miRNA) are small, non-coding RNAs that regulate gene expression post-transcriptionally. Among them, miRNA-34a targets and regulates p53 pathways to induce cell apoptosis and cell-cycle arrest in tumor cells. Three ELP variants were constructed in this study, including Tat-A86 with the sequence Tat-(AP1-V_5_)_16_-[(V_3_G_3_A_1_)_3_V_7_]_3_, A86 with the sequence (AP1-V_5_)_16_-[(V_3_G_3_A_1_)_3_V_7_]_3_, and Tat-E_60_ with the sequence Tat-(V_3_G_3_A_1_)_16_(V_7_)_5_. V, G, and A represent the guest residues in the ELP pentapeptide repeat sequence (VPGXG)_n_. Each construct incorporated cationic short peptides—Tat and/or AP1—that enabled electrostatic condensation of miRNA. Upon miRNA loading, these ELPs self-assembled into nanocomplexes ranging from 31 to 145 nm at 37 °C and effectively protected miRNA from RNase-mediated degradation. Among the three constructs, miRNA-34a/Tat-A86 nanoparticles, which contained both Tat and AP1, exhibited a 3-4-fold greater ability to induce apoptosis in LLC murine lung cancer cells compared to miRNA-34a/Tat-E60 or miRNA-34a/A86, despite showing similar cellular uptake efficiencies. Consistently, in LLC tumor-bearing mice, miRNA-34a/Tat-A86 nanoparticles achieved the highest therapeutic efficacy. No systemic toxicity was detectable across all formulations.

## 4 Challenges and future directions

ELP micelles and micelle-like nanoparticles represent a promising class of drug delivery systems characterized by low immunogenicity, high biocompatibility, and exceptional tunability. They have been successfully employed in preclinical models for cancer therapy, immunization, and treatment of diseases such as wound healing and ocular disorders. Beyond micelles, ELP monomers, which are typically 6–10 nm, are used to extend the circulation time of conjugated drugs, and ELP depots, which are formed after subcutaneous injection to enable controlled and sustained drug release, have also been extensively investigated for disease therapy and tissue repair. However, no ELP–based formulations have yet received FDA approval. Clinical translation of ELP-based formulations faces several key challenges. First, ELPs represent a relatively new class of genetically engineered biomaterials; therefore, extensive preclinical evaluation is required to establish their safety, pharmacokinetic behavior, and manufacturing reproducibility prior to human use (Meyer and Chilkoti, 1999; Mackay and Chilkoti, 2008). Second, large-scale production of long-chain ELPs—particularly constructs exceeding ∼40 pentapeptide repeats that are often needed for stable micelle formation—can be technically demanding. Recombinant expression of long-chain ELPs in bacterial systems may suffer from genetic instability, reduced yields, and host cell stress–associated degradation. Although ELPs can be enriched efficiently using ITC or organic extraction, these approaches alone may not consistently achieve the purity standards required for clinical applications. In addition, bacterial expression introduces the risk of endotoxin contamination, necessitating validated downstream processes to meet clinically acceptable endotoxin limits (Mackay and Chilkoti, 2008). Finally, the thermo-responsive phase-transition behavior and high tunability of ELPs—while advantageous for controllable self-assembly and drug delivery—can also introduce practical risks, including unexpected aggregation driven by sensitivity to temperatureand ionic strength *in vivo*. Collectively, these factors can slow the clinical translation of ELP-based nanomedicines compared to more established polymer-based delivery systems.

Currently, several ELP-based formulations developed by PhaseBio Pharmaceuticals Inc. for treating diabetes, heart failure, and pulmonary arterial hypertension are currently undergoing clinical trials, with some having completed safety, tolerability, and pharmacokinetic evaluations (Inc. PP, 2022a; ClinicalTrials.gov., 2018; ClinicalTrials.gov., 2019; ClinicalTrials.gov., 2020; ClinicalTrials.gov., 2022b). ELP-based drug delivery systems are well suited for peptide delivery as ELPs can be recombinantly fused to peptide drugs or ligands. Accordingly, ELP-based formulations evaluated in clinical trials have primarily focused on ELP-peptide fusions, such as PB1023 (an ELP–glucagon-like peptide-1 [GLP-1] fusion) for the treatment of type 2 diabetes, and PB1046 (an ELP–vasoactive intestinal peptide [VIP] fusion) for pulmonary arterial hypertension.

Despite these advances, designing clinically translatable ELP micelles for broader drug delivery applications remains challenging. First, the T_t_ of ELPs is sensitive to multiple parameters, including the hydrophobicity of the conjugated drug, environmental pH, ionic strength, and polymer concentration. While this tunable property enables the design of responsive delivery systems, it can also compromise stability. For instance, diblock ELPs that self-assemble into micelles at 37 °C may revert to monomers at 4 °C or room temperature, making encapsulated drugs vulnerable to degradation during storage and necessitating temperature-controlled injection to maintain micellar integrity. To address this, strategies such as chemical crosslinking and incorporation of hydrophobic moieties (e.g., peptides or drugs) have been developed to increase micelle stability. Nevertheless, micelle disassembly upon dilution in blood or exposure to variable tissue environments remains a concern because these changes may result in unpredictable *in vivo* behavior, including deviations between expected and actual biodistribution and therapeutic efficacy. Second, recombinant production yield of ELPs in *E. coli* is strongly influenced by guest residue composition, chain length, and the hydrophobicity of conjugated peptide or protein drugs. Hydrophilic ELPs with high T_t_ often show low purification efficiency *via* ITC due to incomplete phase transitions under practical temperature and salt conditions, whereas highly hydrophobic ELPs tend to form insoluble inclusion bodies, reducing soluble yield and bacterial growth rates. Moreover, endotoxin removal from *E. coli*-derived ELPs remains another barrier to their clinical translation.

In the future, computationally guided design of drug-loaded ELP micelles—including machine learning (ML)-based approaches— could markedly improve prediction of their physicochemical properties, including T_t_, size, shape, and assembly–disassembly dynamics in different tissue environments (Hoseini et al., 2023; Chan et al., 2025). Integrating computational modeling with experimental data could also enhance our ability to forecast their *in vivo* performance, such as biodistribution, circulation half-life, and drug release kinetics. Furthermore, future mechanistic and modeling studies should aim to establish rational design principles for developing stable, targeted, and stimulus-responsive ELP micelles by defining the optimal balance between hydrophobic and hydrophilic segments, exploring broader guest residue combinations, and conjugating ELPs with diverse polymers to achieve improved structural stability and functional versatility.
